# Metabolic Reprogramming of *NSUN2*-Mediated m^5^C Modification Promotes the Progression of Hepatocellular Carcinoma by Restricting Natural Killer Cell-Mediated Cytotoxicity

**DOI:** 10.34133/cancomm.0043

**Published:** 2026-08-31

**Authors:** Shengwei Mao, Yuan Fang, Jun Gao, Jiafeng Chen, Zhiqi Guan, Xuhui Zhao, Jinglei Wu, Xiaoling Wu, Guiqi Zhu, Xiangyu Zhang, Qianfu Zhao, Rui Yang, Yi Wang, Tianhao Chu, Yichao Bu, Jialu Fu, Hongxu Li, Zheng Tang, Yinghong Shi, Jian Zhou, Jia Fan, Jinling Jiang, Weiren Liu

**Affiliations:** ^1^ Department of Liver Surgery and Transplantation, Zhongshan Hospital, Fudan University, Shanghai, P. R. China.; ^2^ Research Unit of Liver Cancer Recurrence and Metastasis, Chinese Academy of Medical Sciences, Beijing, P. R. China.; ^3^ Key Laboratory of Carcinogenesis and Cancer Invasion, Ministry of Education, Shanghai, P. R. China.; ^4^ Department of General Surgery (Thyroid and Breast), Zhongshan Hospital, Fudan University, Shanghai, P. R. China.; ^5^ Department of General Surgery, Shanghai Geriatric Medical Center (Zhongshan Hospital Minhang Campus), Fudan University, Shanghai, P. R. China.; ^6^ College of Clinical Medicine, Shanghai Medical College, Fudan University, Shanghai, P. R. China.; ^7^ Department of Colorectal Surgery, Zhongshan Hospital, Fudan University, Shanghai, P. R. China.; ^8^ Liver Cancer Institute, Zhongshan Hospital, Fudan University, Shanghai, P. R. China.; ^9^ Department of Oncology, Ruijin Hospital, Shanghai Jiao Tong University School of Medicine, Shanghai, P. R. China.

## Abstract

**Background:** Hepatocellular carcinoma (HCC) remains a leading cause of cancer-related mortality, with resistance to immunotherapy posing a major clinical challenge. Natural killer (NK) cells exhibit impaired infiltration and cytotoxicity in HCC; however, the mechanisms underlying NK cell-mediated immune evasion are still poorly understood. This study investigated how NOP2/Sun RNA methyltransferase 2 (NSUN2), a 5-methylcytosine (m^5^C) RNA methyltransferase, induces metabolic reprogramming and immunosuppression to drive HCC progression. **Methods:** We conducted a genome-wide CRISPR screen in HCC cells cocultured with NK cells. To delineate the downstream mechanisms, we integrated profiling of the m5C epitranscriptome, transcriptome, and chromatin landscape with metabolic characterization. The impact of *NSUN2* on histone lactylation and programmed cell death 1 ligand 1 (PD-L1) transcription was further investigated. Functional assays in vitro and in vivo using syngeneic murine models and pharmacological inhibition validated these findings. Clinical relevance was assessed using patient tissues, The Cancer Genome Atlas dataset, and immunotherapy cohorts. **Results:** Genome-wide CRISPR screening in HCC cell–NK cell coculture models identified *NSUN2* as a key suppressor of NK cell-mediated cytotoxicity. Mechanistically, NSUN2-mediated RNA m^5^C modification enhanced the messenger RNA stability and expression of glycolytic enzymes, including enolase 1 (*ENO1*), pyruvate kinase M1/2 (*PKM*), and lactate dehydrogenase A (*LDHA*), thereby increasing lactate production. Accumulated lactate promoted histone H3 lysine 18 lactylation (H3K18la), which enhanced chromatin accessibility at the *CD274* (encoding PD-L1) promoter and recruited signal transducer and activator of transcription 3 (STAT3) to drive PD-L1 expression, ultimately inhibiting NK cell-mediated cytotoxicity. Clinically, high NSUN2 expression was associated with elevated PD-L1 levels, poor prognosis, and immunotherapy resistance in patients with HCC. In vivo, *NSUN2* knockout increased NK cell infiltration and suppressed tumor growth, while the STAT3 inhibitor TTI-101 combined with anti-PD-L1 therapy enhanced NK cell cytotoxicity and inhibited HCC progression. **Conclusions:** Our data demonstrated that NSUN2 drove immune evasion in HCC by coupling m^5^C-dependent glycolytic reprogramming with H3K18la-mediated epigenetic activation of PD-L1. These findings suggest that NSUN2 could represent a critical nexus between m^5^C RNA methylation and immunosuppression, providing a therapeutic rationale for combination immunotherapy in HCC.

## Background

The incidence of hepatitis B virus-related hepatocellular carcinoma (HCC) has gradually decreased due to the decline in hepatitis B virus infection; however, the mortality rate remains elevated, primarily due to late-stage diagnosis and limited therapeutic options [1]. Although immunotherapy advancements, particularly those involving immune checkpoint inhibitors (ICIs) targeting programmed cell death protein 1 (PD-1) and programmed cell death 1 ligand 1 (PD-L1), have improved outcomes in patients with unresectable HCC, resistance to immunotherapy remains a considerable challenge [2,3]. Natural killer (NK) cells are critical components of innate antitumor immunity and exhibit potent cytotoxic activities against malignant cells [4,5]. However, HCC tumors often evade NK cell surveillance via immunosuppressive mechanisms, including reduced NK cell infiltration and impaired cytotoxicity [6–8]. Unlike T cells, which rely on major histocompatibility complex (MHC)-restricted presentation, NK cells are regulated by the interplay between surface-activating and inhibitory receptors [9]. Notably, immune checkpoint activation in NK cells impairs their antitumor function [10]. Deciphering the molecular drivers of NK cell immunosuppression in HCC holds promise for developing precision immunotherapies to overcome resistance mechanisms.

Emerging evidence suggests that such immune evasion is orchestrated by the interplay between epigenetic and metabolic alterations within the tumor microenvironment [11–14]. RNA chemical modification is an important form of epitranscriptomic regulation, with 5-methylcytosine (m^5^C) serving as a key posttranscriptional regulator influencing RNA stability, translation, and metabolic reprogramming [15]. NOP2/Sun RNA methyltransferase 2 (*NSUN2*) is a conserved m^5^C methyltransferase involved in tumor progression [16–18]; however, its role in modulating antitumor immunity remains unexplored. Specifically, it is unknown whether *NSUN2*-mediated RNA methylation contributes to the immunosuppressive phenotype of HCC, potentially by reshaping tumor cell metabolism. Metabolic reprogramming, such as enhanced glycolysis, fuels tumor growth and generates oncometabolites such as lactate, which can epigenetically suppress immune responses [19,20]. Notably, lactate can serve as a precursor for epigenetic modifications [21,22]. Histone posttranslational modifications (PTMs) are associated with various metabolites, including crotonic acid (crotonylation), succinic acid (succinylation), and lactate (lactylation) [22,23]. Histone lactylation is involved in tumor metabolic regulation, epigenetic regulation, and signal transduction [24]. Crucially, recent studies indicate that histone lactylation can drive the expression of immunosuppressive genes, providing a potential mechanistic link from glycolysis to immune checkpoint regulation in malignancies [25–29].

Despite the emerging importance of RNA methylation and histone lactylation in cancer biology, the potential crosstalk between *NSUN2*-mediated m^5^C modification and NK cell-mediated immune escape in HCC remains to be elucidated. Therefore, the primary objective of this study was to determine whether *NSUN2* drives an immunosuppressive tumor microenvironment by reshaping metabolic pathways. Specifically, we hypothesized that *NSUN2*-mediated RNA methylation facilitates metabolic reprogramming toward glycolysis, leading to an accumulation of lactate that promotes histone lactylation and subsequent immune evasion. To test this, we employed a combination of epigenomic and transcriptomic techniques, metabolic characterization, as well as in vitro and in vivo immunological assays to investigate the regulatory effect of *NSUN2* on NK cell activity. By defining the mechanistic links within the *NSUN2*/lactate/histone lactylation axis, this study aimed to identify therapeutic targets to overcome immunotherapy resistance in HCC.

## Materials and Methods

### Patients and clinical tissue specimens

Paired snap-frozen human HCC tumor tissues and adjacent normal tissues (≥2 cm from the tumor edge) were obtained from 30 patients undergoing surgical liver resection at Zhongshan Hospital, Fudan University (Shanghai, China) between December 2023 and August 2024. The inclusion criteria were (a) histopathologically confirmed primary HCC, and (b) no prior anticancer therapy (including chemotherapy, radiotherapy, targeted therapy, or immunotherapy) prior to sample collection. Additionally, an immunotherapy cohort of 30 patients treated with anti-PD-1 immunotherapy at Zhongshan Hospital between May and July 2024 was included. This cohort included 15 responders and 15 nonresponders, from whom biopsy or surgical resection specimens were obtained. The HCC tissue microarray (TMA) cohort was constructed using samples from 355 HCC patients who underwent liver resection at Zhongshan Hospital between 2006 and 2008. Following surgery, these patients were assessed using computed tomography or abdominal magnetic resonance imaging scans every 6 months during the first 2 years, followed thereafter by abdominal ultrasound at 3-month intervals. Serum alpha-fetoprotein levels and liver function were also assessed at 3-month intervals. Follow-up continued until December 2013. None of the patients in the TMA cohort had received any preoperative anticancer interventions such as radiofrequency ablation, anhydrous alcohol injection, transarterial chemoembolization, or immunotherapy. All samples were histopathologically confirmed as HCC. Peripheral blood samples were obtained from 8 healthy donors during the same period for primary NK cell isolation. Written informed consent was obtained from all patients and healthy donors. A summary of the clinical and pathological characteristics of the enrolled HCC patients is provided in Table S1.

### Cell culture

The human hepatoma cell lines MHCC-97H (RRID: CVCL_4972) and Huh7 (RRID: CVCL_0336), as well as the murine hepatoma cell line Hepa1-6 (RRID: CVCL_0327), were purchased from the Shanghai Biological Cell Bank of the Chinese Academy of Sciences (Shanghai, China). The cells were cultured in Dulbecco’s modified Eagle’s medium (#10564011, Gibco) supplemented with 10% fetal bovine serum (A5256501, Gibco) and 1% penicillin-streptomycin-neomycin (#15640055, Gibco) in a cell culture incubator containing 5% CO_2_ at 37 °C. All cell lines were authenticated by short tandem repeat profiling and tested negative for mycoplasma contamination. Human NK cells were isolated from the peripheral blood of healthy donors via negative selection using the EasySep Direct Human NK Cell Isolation Kit (#19665, StemCell Technologies). Isolated NK cells were cultured and expanded using the ImmunoCult NK Cell Expansion Kit (#100-0711, StemCell Technologies) according to the manufacturer’s instructions.

### Cell treatment

To investigate the impact of glycolytic metabolism on histone lactylation and PD-L1 expression, MHCC-97H cells were treated with various glycolysis inhibitors or metabolites. Specifically, cells were incubated with 2-deoxy-D-glucose (2-DG; 10 mmol/l; HY-13966, MedChemExpress), sodium dichloroacetate (DCA; 20 mmol/l; HY-Y0445A, MedChemExpress), or oxamate (20 mmol/l; HY-W013032A, MedChemExpress). To mimic the lactate-rich tumor microenvironment, cells were treated with exogenous sodium L-lactate (20 mmol/l; L7022, Sigma-Aldrich). For the combinatorial therapeutic analysis and NK cell cytotoxicity assays, MHCC-97H cells were treated with the signal transducer and activator of transcription 3 (STAT3) inhibitor TTI-101 (10 μmol/l; HY-112288, MedChemExpress), the anti-PD-L1 antibody (10 μg/ml; #374502, BioLegend), or a combination of both. Dimethyl sulfoxide (HY-Y0320C, MedChemExpress) served as the vehicle control. Following treatment, cells were harvested for Western blotting, flow cytometry, or coculture assays as described below.

### Animals and tumor models

C57BL/6 and nonobese diabetic/severe combined immunodeficiency (NOD/SCID) mice (6 to 8 weeks old) were purchased from the Shanghai Laboratory Animal Center of the Chinese Academy of Sciences (Shanghai, China). Experimental operations and animal husbandry were performed at the Department of Laboratory Animal Science of the Fudan University Fenglin Campus. Mice were housed under specific pathogen-free conditions in the animal facility of the Department of Laboratory Animal Science, Fudan University. The environment was strictly controlled with a 12-h light/dark cycle, constant temperature (22 ± 2 °C) and humidity (50% ± 10%), with autoclaved food and water provided ad libitum. To minimize suffering, humane endpoints were strictly defined and monitored, including severe dyspnea, prolonged lethargy, inability to reach food or water, a tumor burden exceeding 15% of body weight, or other signs of severe distress. Upon reaching these humane endpoints or at the study’s conclusion, mice were euthanized via cervical dislocation by trained personnel, and death was confirmed prior to tissue collection.

Various mouse tumor models were established as follows. For the subcutaneous tumor model, Hepa1-6 cells (1 × 10^6^) were resuspended in 100 μl of phosphate-buffered saline (PBS; G4207, Servicebio) and injected subcutaneously into the right posterior flank of the mice. For the orthotopic tumor model, mice were anesthetized with isoflurane. Hepa1-6 cells (1 × 10^5^) were resuspended in 15 μl of PBS, mixed with 15 μl of basement membrane matrix (#354248, Corning), and injected into the left liver lobe using a microinjector, followed by wound suturing. For the spontaneous tumor model, an HCC model was established via hydrodynamic tail vein injection (HDTVi). In brief, plasmids [20 μg each of pT3-EF1A-MYC-IRES-Luc, pT3-EF1a-N90-β-catenin, and pCMV-SB13 encoding Sleeping Beauty (SB) transposase] were suspended in 2 ml of 0.9% sterile saline solution. The solution was administered by injection into the lateral tail vein of the mouse over a period of approximately 5 to 7 s.

### Bioluminescence imaging

Mice were anesthetized via isoflurane inhalation and subsequently administered 150 μl of D-luciferin sodium salt solution (20 mg/ml; HY-12591, MedChemExpress) via intraperitoneal injection. Five minutes postinjection, bioluminescence images were acquired using the In Vivo Imaging System Spectrum (PerkinElmer). The bioluminescence signal intensity within regions of interest was quantified using Living Image software (version 4.4; PerkinElmer) to evaluate tumor burden.

### In vivo cytotoxicity assay

To evaluate the cytotoxic activity of NK cells in vivo, luciferase-labeled MHCC-97H (MHCC-97H-Luc) cells with *NSUN2* knockout (KO) or control (2 × 10^5^ cells per mouse) were inoculated into NOD/SCID mice via intraperitoneal injection. Seven days postinoculation, human primary NK cells (2 × 10^6^ cells per mouse) were administered via intraperitoneal injection. Bioluminescence imaging (BLI) was performed to monitor the engraftment and progression of tumor cells.

### In vivo therapeutic protocols

Mice were randomly assigned into groups (*n* = 6 per group) and treated with vehicle, TTI-101 (STAT3 inhibitor), anti-PD-L1, or the combination of both. TTI-101 (50 mg/kg; HY-112288, MedChemExpress) was dissolved in olive oil (O1514, Sigma-Aldrich) containing 5% dimethyl sulfoxide and administered once daily via intraperitoneal injection. Anti-PD-L1 antibody (10 mg/kg; clone 10F.9G2, A2115, Selleck) was diluted in sterile PBS and administered via intraperitoneal injection every 3 days. Treatment was initiated after tumors reached a consistent bioluminescence threshold: on day 10 for orthotopic Hepa1-6 liver tumors, and on day 7 following HDTVi in the spontaneous model.

### Immune cell depletion

NK cells were depleted using an anti-NK1.1 antibody (clone PK136, A2114, Selleck) administered at a dose of 200 μg via intraperitoneal injection every 3 days, starting 1 day prior to orthotopic transplantation. CD8^+^ T cells were depleted using an anti-mouse CD8ɑ antibody (clone 2.43, A2102, Selleck) at the same dose and via the same route following the same schedule. A mixture of Mouse IgG2a (A2117, Selleck) and Rat IgG2b (A2116, Selleck) served as the isotype control.

### NK cell cytotoxicity assay in vitro

MHCC-97H and Huh7 hepatoma cells were seeded into 96-well plates at a density of 5 × 10^4^ cells/well and incubated for 24 h to facilitate adhesion. Human peripheral blood-derived NK cells were then added at effector-to-target (E:T) ratios of 1:1, 2:1, and 5:1. The coculture was maintained under standardized conditions (37 °C, 5% CO_2_, and a humidified atmosphere) for 4 h. Target cell spontaneous release, effector cell spontaneous release, and target cell maximum release controls were set up simultaneously. Cytotoxicity was assessed using a lactate dehydrogenase (LDH) Cytotoxicity Assay Kit (40209ES76, Yeasen) according to the manufacturer’s protocol. Briefly, for the target cell maximum release wells, 15 μl of LDH release reagent was added 1 h prior to the end of incubation. Upon completion of the coculture, the plates were centrifuged at 400 × *g* for 5 min at room temperature. Subsequently, 120 μl of supernatant from each well was transferred to a fresh 96-well plate. The LDH detection working solution (60 μl) was added to each well and incubated in the dark for 30 min at room temperature. Absorbance was measured at 490 nm, with a reference wavelength of 650 nm, using a microplate reader (Varioskan LUX, Thermo Fisher Scientific).

### Scanning electron microscopy and transmission electron microscopy

NK cells were isolated from tumor cell cocultures via flow cytometry. For scanning electron microscopy (SEM), the sorted cells were fixed with a mixture of 0.5% glutaraldehyde (#49629, Sigma-Aldrich) and 4% paraformaldehyde (60536ES60, Yeasen), whereas for transmission electron microscopy (TEM), fixation was performed using 2.5% glutaraldehyde. Following primary fixation, all samples were postfixed with 1% osmium tetroxide (#201030, Sigma-Aldrich) and dehydrated through a graded ethanol series. SEM specimens were subsequently substituted with isoamyl acetate, critical-point dried, sputter-coated with gold-palladium, and examined using a SU8010 scanning electron microscope (Hitachi). Conversely, TEM samples were en bloc stained with uranyl acetate, embedded in epoxy resin, and sectioned into ultrathin slices. These sections were further contrasted with lead citrate and uranyl acetate and examined using a JEM-1400 Flash transmission electron microscope (JEOL) operating at 120 kV.

### Quantitative real-time polymerase chain reaction

Total RNA was extracted from cultured cells and frozen tissues using TRIzol Reagent (#15596026, Thermo Fisher Scientific). The concentration and purity of the RNA were determined using a NanoDrop 2000 spectrophotometer (Thermo Fisher Scientific). Complementary DNA (cDNA) was synthesized from 1 μg of total RNA using the HiScript III 1st Strand cDNA Synthesis Kit (R312-01, Vazyme). Quantitative PCR was performed using ChamQ Universal SYBR quantitative PCR (qPCR) Master Mix (Q221-01, Vazyme) on a CFX96 Real-time System (Bio-Rad). The thermal cycling conditions were as follows: initial denaturation at 95 °C for 30 s; followed by 40 cycles of denaturation at 95 °C for 10 s and annealing/extension at 60 °C for 30 s. Relative messenger RNA (mRNA) expression levels were calculated using the 2^−ΔΔCt^ (Ct = threshold cycles) method and normalized to the endogenous control *ACTB*. Primer sequences are listed in Table S2.

### Cell transfection

MHCC-97H cells were serum-starved in serum-free Opti-MEM medium (#31985062, Thermo Fisher Scientific) for 12 h before transfection. Small interfering RNAs (siRNAs) targeting *PUS7*, *SETDB1*, *BCL7A*, *FNBP1L*, *NSUN2*, *PAIP1*, *DARS2*, *SLC35F5,* and *P300* were synthesized by Tsingke Biotech (Shanghai, China). For overexpression (oe) assays, the full-length cDNA sequences of human *NSUN2* and *CD274* (PD-L1) were cloned into the pcDNA3.1 vector (Tsingke Biotech). Transfections were performed according to the manufacturer’s protocol using Lipofectamine 3000 (L3000001, Thermo Fisher Scientific). Cells were harvested 48 h posttransfection for subsequent analysis. The siRNA sequences are provided in Table S3.

### Lentiviral transduction

Short hairpin RNAs (shRNA) targeting *STAT3*, *ELF1*, and *CD274* were synthesized by General Biosystems (Anhui, China). Lentiviral particles were generated using a Lentiviral Packaging Kit (41102ES10, Yeasen) and used to transduce target cells for 24 h. Transduced cells were selected with 1 μg/ml puromycin (60209ES10, Yeasen) for 7 days, followed by maintenance in medium containing 0.4 μg/ml puromycin prior to subsequent quantitative real-time polymerase chain reaction (qRT-PCR) and Western blotting analysis to verify interference efficiency. The shRNA sequences are provided in Table S3.

### Site-directed mutagenesis

The full-length coding sequence of human *NSUN2* (NCBI RefSeq: NM_017755.6) was PCR-amplified and cloned into the pLenti-CMV-GFP-Neo lentiviral vector (General Biosystems) to generate the wild-type construct, pLenti-NSUN2(WT). To investigate the functional roles of specific catalytic residues, cysteine-to-alanine substitutions at C271 and C321 (C271A and C321A, respectively) were introduced using overlap extension PCR. Point mutations were designed to convert the target codons from TGT/TGC to GCT. The resulting mutant fragments were subsequently subcloned into the pLenti-CMV-GFP-Neo backbone. All constructs were verified by Sanger sequencing to ensure the presence of intended mutations and the absence of any unintended mutations. The verified plasmids were then used for lentiviral particle production as described previously.

### Genome-wide CRISPR screening

CRISPR-associated protein 9 (Cas9)-expressing MHCC-97H cells were generated by transducing cells with the pLV-Cas9-Neo lentivirus (General Biosystems) followed by selection with G418. The expression of the Cas9 protein was verified via Western blotting. The Human GeCKOv2 Library B, a pooled library containing 65,383 single-guide RNAs (sgRNAs) targeting 19,050 genes, was packaged into lentivirus and used to transduce Cas9-expressing MHCC-97H cells at a multiplicity of infection of 0.3, followed by puromycin selection for 1 week. Engineered MHCC-97H cells were cocultured with human peripheral blood-derived NK cells at an E:T ratio of 1:2. After 36 h of coculture, NK cells and cellular debris were removed by washing, and surviving tumor cells were collected. Control cells (without NK cell coculture) were harvested in parallel for comparison. Genomic DNA was extracted from both the experimental and control groups. To ensure adequate library representation, the sgRNA-encoding regions were amplified by PCR using specific primers flanking the variable region. The resulting amplicons were purified and used to construct sequencing libraries compatible with the Illumina platform. The libraries were quantified using Qubit 3.0 and subsequently analyzed using an Agilent 2100 Bioanalyzer. To ensure high-quality screening standards, the experiment was performed with 3 independent biological replicates maintaining a library coverage of >500× cells per sgRNA (sequencing depth > 400 reads/sgRNA; Gini index 0.18). Sequencing data were processed using MAGeCK (version 0.5.9, https://github.com/liulab-dfci/MAGeCK), which performed guide RNA (gRNA) and gene count normalization, followed by a comparative analysis of differential gRNA and gene abundance between the experimental and control groups.

### Western blotting

Total protein from tissues and cells was extracted using radioimmunoprecipitation assay lysis buffer (P0013B, Beyotime) supplemented with 1 mmol/l phenylmethanesulfonyl fluoride protease inhibitor (ST507, Beyotime). Protein concentrations were quantified using a bicinchoninic acid assay kit (P0012, Beyotime). Equal amounts of protein samples were separated by 10% to 12.5% sodium dodecyl sulfate-polyacrylamide gel electrophoresis and transferred onto polyvinylidene difluoride membranes. After blocking with 5% nonfat milk in Tris-buffered saline with Tween-20 for 1 h at room temperature, the membranes were incubated with primary antibodies overnight at 4 °C. The membranes were then washed and incubated with horseradish peroxidase-conjugated secondary antibodies for 2 h at room temperature. Immunoblots were visualized using the West Dura Extended Duration Substrate kit (#34075, Thermo Fisher Scientific) and a chemiluminescence imaging system (#5200, Tanon). For the analysis of site-specific histone lactylation, histone H3 lysine 18 lactylation (H3K18la), histone H3 lysine 14 lactylation (H3K14la), and histone H3 lysine 9 lactylation (H3K9la) were examined by Western blotting using corresponding antibodies. Antibody information is provided in Table S4.

### Immunofluorescence staining

For multiplexed immunofluorescence (IF) analysis, formalin-fixed, paraffin-embedded tissue sections (4 μm thick) were baked at 60 °C for 1 h, followed by deparaffinization and rehydration. Antigen retrieval was performed by boiling sections in ethylenediaminetetraacetic acid buffer (pH 8.0; C0196, Beyotime). Sections were then blocked and permeabilized with 3% bovine serum albumin (ST2254, Beyotime) in PBS containing 0.25% Triton X-100 (P0096, Beyotime) for 1 h at room temperature, followed by incubation with primary antibodies overnight at 4 °C. After 3 washes with PBS, fluorophore-conjugated secondary antibodies were applied for 1 h at room temperature in the dark. Nuclei were counterstained with 4′,6-diamidino-2-phenylindole (DAPI, 5 μg/ml; G1012, Servicebio) for 10 min, and the slides were mounted using an anti-fade mounting medium. Fluorescent images were captured using a confocal laser scanning microscope (LSM880, Zeiss) equipped with appropriate filter sets. Antibody information is detailed in Table S4.

### RNA m^5^C dot blot assay

Total RNA was extracted from MHCC-97H cells using TRIzol reagent, and mRNA was isolated from the total RNA using the Hieff Next Generation Sequencing mRNA Isolation Master Kit (12629ES24, Yeasen). The isolated mRNA was denatured at 65 °C for 2 min and immediately chilled on ice for 5 min. Serial dilutions of mRNA (50, 100, and 200 ng) were spotted onto a Hybond-N^+^ membrane (GERPN203B, Merck), followed by crosslinking with ultraviolet light for 5 min. The membrane was blocked with 5% nonfat milk in PBS with Tween-20 (G2157, Servicebio), incubated with m^5^C antibody (1:1,000; ab214727, Abcam) at 4 °C overnight, and further incubated with a horseradish peroxidase-conjugated secondary antibody at room temperature for 1 h. Signals were visualized using a chemiluminescence imaging system. To verify equal loading, the membrane was stained with 0.1% methylene blue (G1301, Solarbio) and washed with distilled water. Antibody information is provided in Table S4.

### Enzyme-linked immunosorbent assay

Total RNA was extracted from tissues and cells using TRIzol Reagent. Global m^5^C RNA methylation levels were quantitatively analyzed using the MethylFlash 5-mC RNA Methylation ELISA (enzyme-linked immunosorbent assay) Easy Kit (P-9009-48, EpiGentek) following the manufacturer’s protocol. Briefly, 200 ng of purified RNA per sample was bound to the assay wells and subjected to sequential washing. Subsequently, a fluorogenic detection substrate was added and incubated at room temperature for 3 min in the dark. Fluorescence intensity was measured using a microplate reader with excitation at 530 nm and emission at 590 nm within 6 min following substrate addition. The relative percentage of m^5^C RNA modification was calculated by parallel quantification against a methylated RNA standard curve, based on the linear relationship between fluorescence signal intensity and methylation levels.

### Assessment of RNA stability

To determine RNA stability, transcription was inhibited by treating the cells with 5 μg/ml actinomycin D (HY-17559, MedChemExpress) in complete culture medium. Cells were harvested at designated time points (0, 3, 6, and 9 h posttreatment) for total RNA isolation using TRIzol Reagent. RNA quantification was performed using qRT-PCR with SYBR Green Master Mix (Q711-02, Vazyme). Relative RNA levels were normalized to the endogenous control *ACTB*, and RNA half-lives (*t*₁/₂) were calculated using nonlinear regression analysis.

### Immunohistochemistry staining

For immunohistochemistry (IHC) experiments, 4-μm formalin-fixed paraffin-embedded tissue sections were deparaffinized, rehydrated, and subjected to antigen retrieval in citrate-based buffer (pH 6.0) using a pressurized decloaking chamber at 95 °C for 15 min. After cooling naturally to room temperature, endogenous peroxidase activity was quenched with 3% H₂O₂ in methanol for 20 min at room temperature. The sections were permeabilized with 0.1% Triton X-100 in PBS for 15 min at room temperature, followed by blocking with 5% normal goat serum for 1 h. Sections were incubated with primary antibodies overnight at 4 °C in a humidified chamber. Detection was performed using the UltraVision Quanto Detection System (Thermo Fisher Scientific), and signals were developed using 3,3′-diaminobenzidine (Y092163, Beyotime). Sections were counterstained with hematoxylin for 30 s, followed by rapid dehydration through a graded ethanol series (70% to 100%) and clearance in xylene. Images were generated using an automatic digital scanning and analysis system (Aperio VERSA) and quantified using Aperio ImageScope software (Leica Biosystems). The IHC score was calculated as staining intensity score (0 to 3) × percentage of positive cells score (0 to 4). Antibody information is provided in Table S4.

### Flow cytometry

For cell surface marker analysis, harvested cells were washed with cold PBS containing 1% bovine serum albumin. The cell suspensions were incubated with fluorophore-conjugated primary antibodies for 30 min at 4 °C in the dark. After washing 3 times with PBS to remove unbound antibodies, the cells were resuspended in PBS. Data acquisition was performed using a flow cytometer (LSRFortessa, BD Biosciences), and data were analyzed using FlowJo software (version 10.8.1, https://www.flowjo.com/). Detailed antibody information is provided in Table S4.

### RNA sequencing

Total RNA was extracted from tumor tissues, MHCC-97H cells, and NK cells using TRIzol Reagent, followed by DNase I (RNase-free) (AM2222, Thermo Fisher Scientific) treatment for 30 min to eliminate genomic DNA contamination. Poly(A)^+^ mRNA was enriched from 1 μg of total RNA using oligo(dT) magnetic beads and fragmented into 100 to 200 base pair (bp) fragments. First-strand cDNA synthesis was performed using SuperScript II Reverse Transcriptase (#18064014, Thermo Fisher Scientific), followed by second-strand synthesis using DNA Polymerase I (M0209S, New England Biolabs). The double-stranded cDNA was purified using AMPure XP beads (A63881, Beckman Coulter), end-repaired, adenylated, and ligated with Illumina-compatible adapters. Library amplification was conducted using 12 cycles of PCR amplification with KAPA HiFi HotStart ReadyMix (KK2602, Roche). Library quality control was performed using an Agilent 2100 Bioanalyzer with High Sensitivity DNA Chips (Agilent Technologies) and quantified using qPCR with the KAPA Library Quantification Kit (KR0405, Roche). Final libraries were sequenced on an Illumina NovaSeq 6000 platform to generate 150-bp paired-end reads.

### Assay for transposase-accessible chromatin with high-throughput sequencing

The assay for transposase-accessible chromatin with high-throughput sequencing (ATAC-seq) libraries were prepared using the ATAC-Seq Library Prep Kit for Illumina (RK21509, ABclonal) following the manufacturer’s protocol with optimized modifications. Briefly, nuclei were isolated by lysing cells with a buffer containing NP-40 and digitonin. Genomic DNA was fragmented using the transposon 5 (Tn5) transposase complex containing Tn5 Transposome, digitonin, Tween-20, and tagment buffer (37 °C for 30 min), followed by purification with AFTMag Next Generation Sequencing DNA Clean Beads (RK20257, ABclonal). Purified DNA fragments were amplified using a PCR master mix and index primers. The PCR conditions were as follows: initial denaturation for 3 min at 95 °C; denaturation for 30 s at 98 °C; 12 to 14 cycles of 15 s at 98 °C and 30 s at 60 °C; 1 min at 72 °C; and a final extension of 5 min at 72 °C. The amplified libraries were size-selected using clean bead purification (0.55× and 1.2× ratios) to retain 150- to 700-bp fragments. Library quantification was performed using the Qubit dsDNA HS Assay Kit (Q32854, Thermo Fisher Scientific), and quality control was verified using an Agilent 2100 Bioanalyzer. Paired-end sequencing (2 × 150 bp) was performed using an Illumina NovaSeq 6000 platform with a minimum depth of 50 million reads per sample. Raw paired-end FASTQ reads were first subjected to quality control and filtering using fastp (version 0.23.4, https://github.com/OpenGene/fastp). The filtered reads were then aligned to the human reference genome (Genome Reference Consortium Human Build 38, GRCh38) using Bowtie2 (version 2.5.4, https://github.com/BenLangmead/bowtie2). The resulting SAM files were converted to BAM format using SAMtools (version 1.19.2, https://github.com/samtools/samtools). Subsequently, the BAM files were sorted, and PCR duplicates were marked with the markdup command in SAMtools, followed by indexing to generate the final processed BAM files. For visualization, BigWig files were generated from the deduplicated BAM files using the bamCoverage function from deepTools (version 3.5.5, https://github.com/deeptools/deepTools). Genome browser tracks were visually inspected in the Integrative Genomics Viewer (version 2.17.4, https://igv.org/). Significantly enriched peaks were identified with MACS3 callpeak (version 3.0.2, https://github.com/macs3-project/MACS), using the processed BAM files as input. Peak annotation was performed using the ChIPseeker R package (version 1.38.0, https://github.com/YuLab-SMU/ChIPseeker). The computeMatrix function from deepTools was used to compute signal matrices across promoter regions. Signal heatmaps were visualized with plotHeatmap function from deepTools.

### Cleavage under targets and tagmentation with high-throughput sequencing

CUT&Tag assays were performed using the CUT&Tag Assay Kit (pAG-Tn5) for Illumina (RK20265, ABclonal) following the manufacturer’s instructions with critical modifications. Briefly, the cells were immobilized on concanavalin A-coated magnetic beads (ConA beads) and permeabilized with 0.05% digitonin. Sequential incubations were performed with the primary antibody (overnight at 4 °C), secondary antibody (1 h at room temperature), and pre-assembled proteinA/G-transposon 5 (pAG-Tn5) transposase complex containing pAG-Tn5 Transposome, digitonin, protease inhibitor cocktail, and Dig-300 Buffer (1 h at room temperature). Tagmentation was initiated by adding 10 mmol/l MgCl₂ and incubating at 37 °C for 1 h. DNA libraries were amplified using Q5 High-Fidelity DNA Polymerase (M0491S, New England Biolabs) with the appropriate cycle numbers determined by input cells. The PCR conditions were as follows: initial incubation for 5 min at 58 °C and 5 min at 72 °C; denaturation for 45 s at 98 °C; 12 to 15 cycles of 15 s at 98 °C and 10 s at 63 °C; and a final extension of 1 min at 72 °C. Purified DNA fragments were size-selected using AMPure XP beads (A63881, Beckman Coulter) and quantified with the Qubit dsDNA HS Assay Kit (Q32851, Thermo Fisher Scientific). Library quality control and quantification were performed using qPCR. Sequencing was performed on an Illumina NovaSeq 6000 platform to generate 150-bp paired-end reads. The processing of raw data was similar to that of ATAC-seq. For motif analysis, de novo discovery of significantly enriched DNA binding motifs within histone mark-associated peak regions was performed with HOMER (version 5.1, http://homer.ucsd.edu/homer/), using the GRCh38 genome sequence as the background model. For transcription factor (TF)-binding motif analysis, H3K18la-marked promoter regions were extracted from cleavage under targets and tagmentation with high-throughput sequencing (CUT&Tag-seq) peak regions and used for downstream motif enrichment analysis [30]. Antibody information is provided in Table S4.

### Methylated RNA immunoprecipitation with next-generation sequencing and MeRIP-qPCR

Total RNA was isolated using TRIzol Reagent. RNA integrity was verified by 1.5% agarose gel electrophoresis (D0137, Beyotime), and quantification was performed using a Qubit 3.0 fluorometer (Thermo Fisher Scientific). RNA was fragmented into 100-nt fragments using RNA Fragmentation Reagents (AM8740, Thermo Fisher Scientific). A 3-μg aliquot of fragmented RNA was reserved as the input control, while the remainder was subjected to immunoprecipitation (IP). Protein A/G magnetic beads were prewashed with IP buffer (150 mmol/l NaCl and 10 mmol/l Tris-HCl, pH 7.5) and incubated with an anti-m^5^C antibody (#39649, Active Motif) for 1 h at 4 °C. After washing, the beads were resuspended in IP buffer and incubated with fragmented RNA for 2 h at 4 °C. The RNA was then eluted and purified. RNA libraries were constructed using the NEBNext Ultra II Directional RNA Library Prep Kit (E7760S, New England Biolabs). Libraries were quality-controlled using an Agilent 2100 Bioanalyzer and sequenced on an Illumina NovaSeq 6000 platform to generate 150-bp paired-end reads. RNA methylation sites were detected using MACS3 (version 3.0.2, https://github.com/macs3-project/MACS). Differentially methylated sites were identified using diffreps (version 1.55.6, https://github.com/shenlab-sinai/diffreps). For MeRIP-qPCR validation, the eluted RNA from both the IP and input fractions was reverse-transcribed using HiScript III RT SuperMix (R323-01, Vazyme). qPCR was subsequently performed using ChamQ Universal SYBR qPCR Master Mix (Q221-01, Vazyme). Antibody information is provided in Table S4.

### Measurement of extracellular acidification rate and oxygen consumption rate

The oxygen consumption rate (OCR) and extracellular acidification rate (ECAR) of HCC cells were measured using an XFe96 Extracellular Flux Analyzer (Agilent Technologies). Briefly, HCC cells were seeded in XFe96 cell culture microplates (103794-100, Agilent) at a density of 5 × 10^4^ cells/well and cultured overnight. On the day of the assay, the culture medium was replaced with Seahorse XF Base Medium supplemented with 2 mmol/l glutamine and 10 mmol/l glucose. Following temperature and pH equilibration in a non-CO₂ incubator at 37 °C for 60 min, measurements were performed according to the manufacturer’s protocol.

### Lactate production measurement

Extracellular L-lactate production was quantified using the L-lactate Colorimetric/Fluorometric Assay Kit (ab65330, Abcam), following the manufacturer’s protocol. Briefly, cells were seeded in 6-well plates and cultured for 24 h. The culture medium was collected and centrifuged at 12,000 × *g* for 5 min at 4 °C to remove cell debris. The supernatants were diluted with Lactate Assay Buffer to fall within the linear range of the standard curve. For the assay setup, 50 μl of Reaction Mix (46 μl lactate assay buffer, 2 μl OxiRed Probe, and 2 μl enzyme mix) was added to standard and sample wells, while 50 μl of Background Control Solution (48 μl Lactate Assay Buffer, 2 μl OxiRed Probe) was added to background wells. After thorough mixing, the plates were incubated at room temperature for 30 min protected from light. Absorbance was measured at 570 nm using a microplate reader. Lactate concentration was normalized to the total protein amount of the cells in the corresponding wells.

### Glucose metabolic flux analysis

Isotope labeling experiments were performed by culturing MHCC-97H cells in glucose-free Dulbecco’s Modified Eagle Medium (#11966025, Thermo Fisher Scientific) supplemented with 10 mmol/l U-^13^C_6_ glucose (CLM-1396, Cambridge Isotope Laboratories). Following a 12-h incubation period, the medium was aspirated, and metabolic activity was immediately quenched by the addition of dry ice-cold 80% methanol (−80 °C). Intracellular metabolites were extracted by scraping the cells and centrifuging the lysates at 16,000 × *g* for 15 min at 4 °C. The clarified supernatants were evaporated to dryness under a nitrogen stream and subsequently reconstituted in the mobile phase. Liquid chromatography–tandem mass spectrometry (LC-MS/MS) analysis was conducted using a Shimadzu Nexera X2 LC-30AD UHPLC system coupled to a Q-Exactive Plus mass spectrometer (Thermo Fisher Scientific).

### Chromatin immunoprecipitation with high-throughput sequencing and ChIP-qPCR

ChIP assays were performed using the Sonication ChIP Kit (RK20258, ABclonal) following the manufacturer’s protocol. Briefly, chromatin was crosslinked with 1% formaldehyde (F8775, Merck), followed by sonication to yield DNA fragments ranging from 200 to 700 bp. Input controls (5% of total chromatin) were collected before IP, and parallel ChIP reactions were performed using target-specific antibodies and negative control IgG. Post-ChIP samples were eluted in ChIP elution buffer and reverse-cross-linked. DNA was purified using the provided spin columns, followed by final centrifugation at 12,000 × *g* for 1 min at room temperature. DNA quantification was performed using a Qubit 4.0 Fluorometer (Thermo Fisher Scientific). For chromatin immunoprecipitation with high-throughput sequencing (ChIP-seq), sequencing libraries were constructed using the ChIP-Seq Library Prep Kit (ABclonal) and sequenced on an Illumina NovaSeq 6000 platform to generate 150-bp paired-end reads. Raw data processing and peak calling were performed using the pipeline described in the ATAC-seq section. For chromatin immunoprecipitation quantitative polymerase chain reaction (ChIP-qPCR) validation, purified DNA was analyzed using ChamQ Universal SYBR qPCR Master Mix. Antibody information is provided in Table S4 and the sequences of primers used for ChIP-qPCR are provided in Table S5.

### Luciferase reporter gene assay

Genomic fragments corresponding to the *CD274* promoter region were cloned into the pGL3-Basic vector (General Biosystems). Cells were seeded into 24-well plates and cultured until they reached approximately 70% to 80% confluence. Subsequently, the cells were cotransfected with the constructed pGL3-CD274 reporter plasmid and the pRL-TK *Renilla* luciferase control plasmid (General Biosystems) using Lipofectamine 3000. Luciferase activities were measured 48 h posttransfection using the Dual-Luciferase Reporter Assay Kit (11402ES60, Yeasen). Relative luciferase activity was calculated by normalizing Firefly luciferase activity to *Renilla* luciferase activity.

### Kyoto Encyclopedia of Genes and Genomes and gene set enrichment analysis

Kyoto Encyclopedia of Genes and Genomes (KEGG) pathway enrichment analysis was conducted to elucidate the biological functions of candidate genes derived from both transcriptomic and epigenomic datasets using the clusterProfiler R package (version 4.10.1, https://bioconductor.org/packages//release/bioc/html/clusterProfiler.html). Gene set enrichment analysis (GSEA) was performed to investigate biological signaling pathways and functional phenotypes associated with *NSUN2* status. For the analysis of RNA sequencing (RNA-seq) data from NK cells cocultured with *NSUN2*-KO or control tumor cells, global gene expression profiles were ranked based on the log2 fold change. The “Natural killer cell-mediated cytotoxicity” gene signature was retrieved from the molecular signatures database to evaluate the functional impairment of NK cells. The normalized enrichment score and false discovery rate were calculated to assess statistical significance.

### Bioinformatic analysis of public datasets

Three independent single-cell RNA-seq datasets (GSE156625, GSE202642, and GSE149614) [31–33] were retrieved from the Gene Expression Omnibus database (https://www.ncbi.nlm.nih.gov/geo/) to characterize the immune landscape of HCC. The raw gene expression matrices were integrated and processed using the Seurat R package (version 4.2.0, https://github.com/satijalab/seurat). Quality control was performed to filter out low-quality cells with high mitochondrial gene content (>10%) or low feature counts (<200). Dimensionality reduction was conducted using principal component analysis and uniform manifold approximation and projection. Major cell types were annotated based on canonical lineage-specific marker genes. The proportion of NK cells within the total immune cell population was quantified and compared between HCC tumor tissues and paired adjacent normal tissues. Furthermore, the expression levels of NK cell-mediated cytotoxicity genes, including perforin 1 (*PRF1*), interferon gamma (*IFNG*), granzyme A (*GZMA*), and granzyme B (*GZMB*), were analyzed to assess NK cell functional states.

The Cancer Genome Atlas-Liver Hepatocellular Carcinoma (TCGA-LIHC) cohort was utilized to obtain gene expression profiles and corresponding clinical information from the TCGA database (https://portal.gdc.cancer.gov/). The RNA-seq read counts were normalized to transcripts per million for subsequent analyses. To identify tumor-intrinsic genes negatively correlated with NK cell infiltration, we utilized a literature-curated NK cell signature (Table S6). Pearson’s correlation analysis was performed between the expression of individual genes and the NK signature score across the TCGA-LIHC cohort. Genes with a significant negative correlation (*R* < −0.3, *P* < 0.05) were intersected with candidate genes identified from the CRISPR screening and a previously published list of genes up-regulated in 19 human tumor types [34]. *NSUN2* expression levels were compared between tumor tissues (*n* = 369) and normal tissues (*n* = 50). For the survival analysis of the multigene signature, a risk score was calculated for each patient by averaging the normalized expression values of the genes included in the signature. Patients were then stratified into high- and low-risk cohorts based on the optimal cutoff value determined by the surv_cutpoint function in the survminer R package (version 0.4.9, https://github.com/kassambara/survminer). Similarly, for single-gene survival analysis, patients were categorized into high- and low-expression groups based on the optimal cutoff value. For the analysis of immunotherapy response, transcriptomic data from the public anti-PD-1-treated HCC cohort ERP117672 was retrieved from the European Nucleotide Archive (https://www.ebi.ac.uk/ena/browser/home) [35]. Patients in this cohort were stratified into responders (*n* = 6), nonresponders (*n* = 29), and nonevaluable samples (*n* = 5) based on their clinical outcomes. The expression levels of *NSUN2* were then quantified and statistically compared between these 2 groups to evaluate the association between *NSUN2* abundance and immunotherapy response.

To identify potential TFs regulating *CD274* (PD-L1) expression, we utilized 4 public databases: hTFtarget (https://guolab.wchscu.cn/hTFtarget/), ENCODE (https://www.encodeproject.org/), GTRD (https://gtrd20-06.biouml.org/), and JASPAR (https://jaspar.elixir.no/). The promoter region of *CD274* (defined as 2 kb upstream of the transcription start site) was scanned for TF binding motifs. Overlapping candidates identified across these databases were selected for further experimental validation.

### Statistical analysis

Statistical analyses were performed using GraphPad Prism (version 10.1.2, https://www.graphpad.com/updates/prism-10-1-2-release-notes) and R software (version 4.3.1, https://cran.rstudio.com/bin/windows/base/old/4.3.1/). Unpaired 2-tailed Student’s *t* tests were used for comparisons between 2 groups. One-way analysis of variance (ANOVA) was used for comparisons among more than 2 groups, and 2-way ANOVA was used for comparisons involving multiple groups with 2 independent variables. Survival analysis was performed using the Kaplan–Meier method, and significance was determined using the log-rank test. The primary endpoint was overall survival (OS). For the TMA cohort, OS was calculated from the date of surgery to the date of death or the last follow-up. For the TCGA-LIHC cohort, OS was calculated from the date of diagnosis to the date of death or the last follow-up. Correlation coefficients were calculated using Pearson’s correlation analysis. A *P* value < 0.05 was considered statistically significant.

## Results

### Genome-wide CRISPR screening identified *NSUN2* as a negative regulator of NK cell-mediated cytotoxicity

**Fig. 1.**
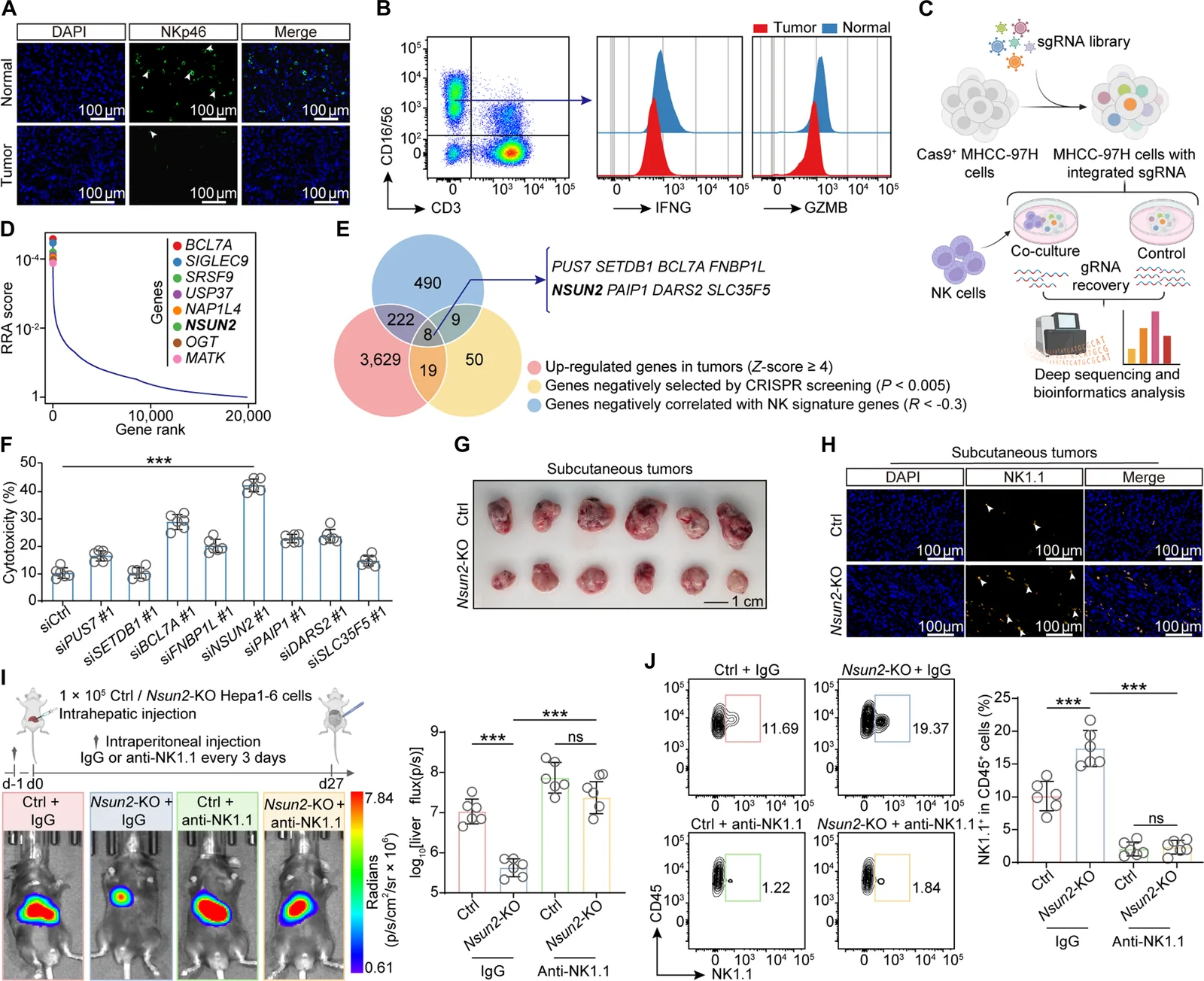
Genome-wide CRISPR screening identified *NSUN2* as a negative regulator of NK cell-mediated cytotoxicity. (A) Representative IF images of NKp46 (green) and DAPI (blue) showing NK cell infiltration in HCC tumor and paired adjacent normal tissues. Scale bars: 100 μm. (B) Representative flow cytometry plots and histograms showing the expression of IFNG and GZMB in NK cells (CD3^−^CD16^+^CD56^+^) from HCC tumor and paired adjacent normal tissues. (C) Schematic illustration of the genome-wide CRISPR screening workflow in MHCC-97H cells cocultured with primary NK cells. (D) Distribution of RRA scores identifying the top 8 candidate genes during CRISPR screening. (E) Venn diagram depicting overlapping genes identified across 3 criteria. (F) NK cell-mediated cytotoxicity against MHCC-97H cells was assessed following siRNA-mediated knockdown of 8 candidate genes. (G) Gross images of subcutaneous tumors derived from control and *Nsun2*-KO Hepa1-6 cells (*n* = 6 per group). (H) Representative IF images of NK1.1 (yellow) and DAPI (blue) showing NK infiltration in control and *Nsun2*-KO Hepa1-6 subcutaneous tumors. Scale bars: 100 μm. (I) Representative BLI showing tumor burden in the indicated groups (left panel), and quantification of bioluminescence intensity (right panel, *n* = 6 per group). Mice were injected with control or *Nsun2*-KO Hepa1-6 cells and treated with IgG or anti-NK1.1 antibody. (J) Representative flow cytometry plots (left panel) and quantification (right panel) of infiltrating CD45^+^NK1.1^+^ cells in the indicated groups. Data are presented as mean ± SD. Statistical significance was determined as follows: 1-way ANOVA was used for (F), and 2-way ANOVA was used for (I) and (J). *P < 0.05; **P < 0.01; ***P < 0.001; ns, not significant. Abbreviations: IF, immunofluorescence; NKp46, natural cytotoxicity triggering receptor 1; DAPI, 4′,6-diamidino-2-phenylindole; NK, natural killer; HCC, hepatocellular carcinoma; IFNG, interferon gamma; GZMB, granzyme B; CRISPR, clustered regularly interspaced short palindromic repeats; Cas9^+^, CRISPR-associated protein 9-expressing; sgRNA, single-guide RNA; gRNA, guide RNA; RRA, robust rank aggregation; siRNA, small interfering RNA; *NSUN2*, NOP2/Sun RNA methyltransferase 2; KO, knockout; BLI, bioluminescence imaging; p/s/cm^2^/sr, photons per second per square centimeter per steradian; p/s, photons per second; NK1.1, natural killer cell lectin-like receptor subfamily B member 1C; IgG, immunoglobulin G; HPF, high-power field; SD, standard deviation; ANOVA, analysis of variance.

Integrative analysis of single-cell RNA-seq datasets from HCC and paired adjacent normal tissues revealed a reduced proportion and diminished cytotoxicity of NK cells within HCC tissues (Fig. S1A to C). These findings were corroborated by IF and IHC staining, demonstrating decreased NK cell infiltration in HCC tissues (Fig. 1A and Fig. S1D to F), and by flow cytometry, showing diminished NK cell effector function (Fig. 1B and Fig. S1G and H). To identify potential tumor-intrinsic regulators of NK cell immunity, we performed a genome-wide human sgRNA library screen in Cas9-expressing MHCC-97H cells cocultured with primary NK cells. We identified 86 candidate genes whose depletion sensitized tumor cells to NK cell-mediated cytotoxicity (Fig. 1C and D and Table S7). Subsequently, we intersected these candidates with 729 genes that were negatively correlated with NK signature genes in the TCGA-LIHC cohort (*R* < −0.3) and 3,878 genes that were up-regulated in 19 human tumor types [34]. Eight overlapping genes were identified across these 3 criteria (Fig. 1E). Validation using siRNA-mediated knockdown of these targets in MHCC-97H cells revealed that *NSUN2* deficiency elicited the most pronounced enhancement of NK cell-mediated cytotoxicity (Fig. 1F). Consistently, analysis of the TCGA-LIHC cohort confirmed a significant negative correlation between *NSUN2* expression and NK signature genes (*R* = −0.33) (Fig. S1I).

To validate these findings in vivo, control and *Nsun2*-KO Hepa1-6 cells were subcutaneously injected into C57BL/6 mice. *Nsun2* KO significantly reduced tumor weight (Fig. 1G and Fig. S1J), which was accompanied by increased NK cell infiltration in *Nsun2*-KO subcutaneous tumors (Fig. 1H and Fig. S1K). Similarly, in a mouse orthotopic liver cancer model, *Nsun2* KO attenuated tumor progression, whereas depletion of NK cells with an anti-NK1.1 antibody reversed this effect (Fig. 1I). Flow cytometry confirmed that *Nsun2* KO increased the infiltration of NK cells (Fig. 1J). Collectively, these results demonstrated that tumor-intrinsic *NSUN2* promoted HCC progression by restricting NK cell-mediated cytotoxicity.

### *NSUN2* deficiency enhanced NK cell-mediated cytotoxicity and reduced PD-L1 expression

**Fig. 2.**
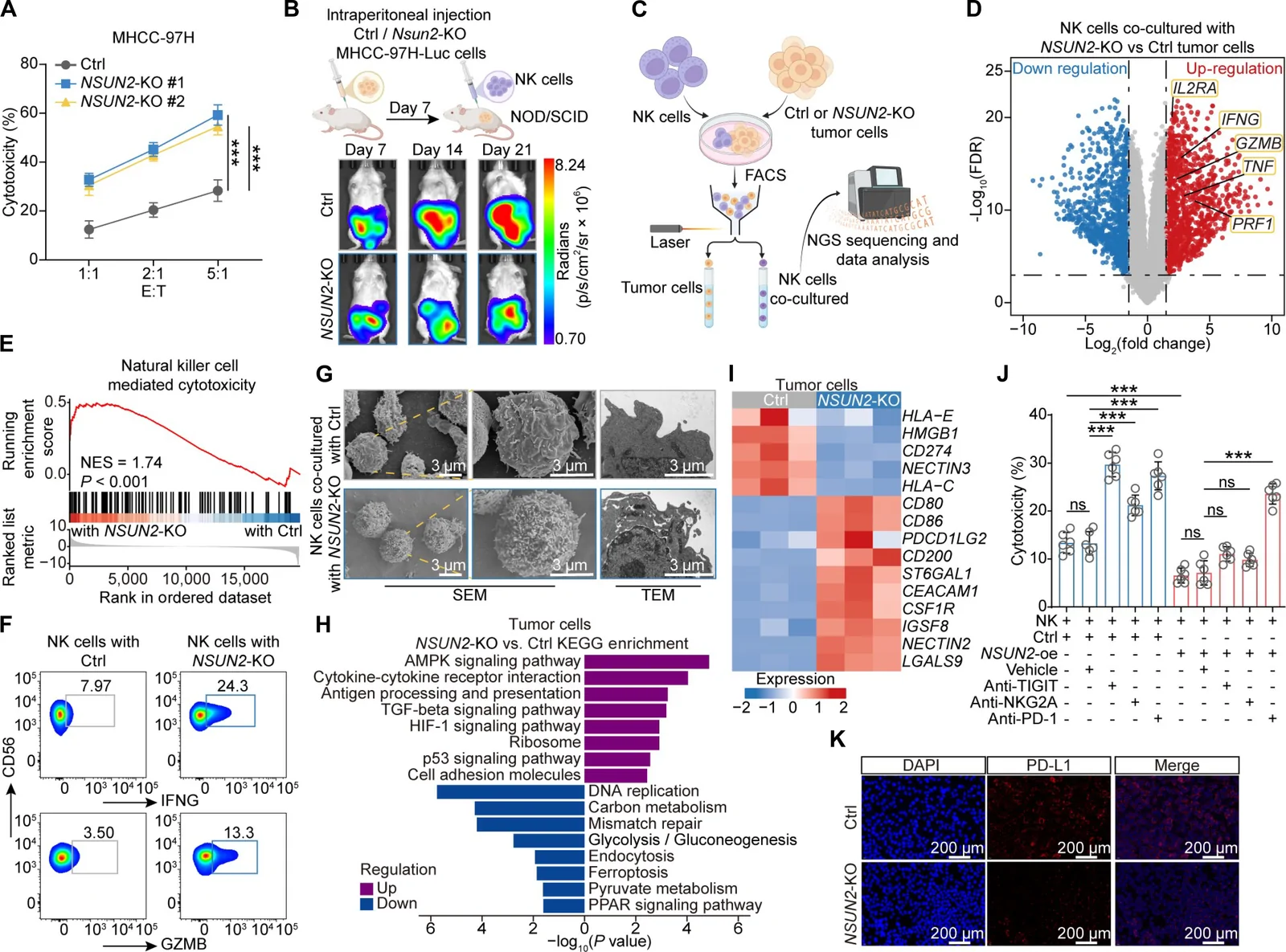
*NSUN2* deficiency enhanced NK cell-mediated cytotoxicity and reduced PD-L1 expression. (A) NK cell-mediated cytotoxicity against control and *NSUN2*-KO MHCC-97H cells at the indicated E:T ratios. (B) Schematic illustration of the in vivo NK cell cytotoxicity assay in the peritoneal cavity of NOD/SCID mice (top panel). Representative BLI images of mice at days 7, 14, and 21 postinjection (bottom panel). (C) Schematic workflow for coculturing NK cells with control or *NSUN2*-KO tumor cells, followed by FACS-based sorting and RNA-seq analysis. (D) Volcano plot showing differentially expressed genes in NK cells cocultured with *NSUN2*-KO versus control tumor cells. (E) GSEA plot showing the enrichment of the “natural killer cell-mediated cytotoxicity” signature in NK cells cocultured with *NSUN2*-KO tumor cells. (F) Representative flow cytometry plots showing the expression of IFNG and GZMB in NK cells after coculture with the indicated tumor cells. (G) Representative SEM (left and middle panels) and TEM (right panel) images of NK cells after coculture with the indicated tumor cells. Scale bars: 3 μm. (H) KEGG pathway enrichment analysis of differentially expressed genes in *NSUN2*-KO versus control tumor cells. (I) Heatmap showing the expression of immune checkpoint-related genes in *NSUN2*-KO versus control tumor cells. (J) NK cell-mediated cytotoxicity against control or *NSUN2*-oe MHCC-97H cells in cocultures treated with the indicated neutralizing antibodies (anti-TIGIT, anti-NKG2A, and anti-PD-1). (K) Representative IF images showing PD-L1 (red) expression and DAPI (blue) in control and *NSUN2*-KO MHCC-97H cells. Scale bars: 200 μm. Data are presented as mean ± SD. Statistical significance was determined as follows: 2-way ANOVA test was used for (A) and (J). *P < 0.05; **P < 0.01; ***P < 0.001; ns, not significant. Abbreviations: NK, natural killer; *NSUN2*, NOP2/Sun RNA methyltransferase 2; KO, knockout; E:T, effector-to-target; NOD/SCID, nonobese diabetic/severe combined immunodeficiency; MHCC-97H-Luc, luciferase-labeled MHCC-97H; BLI, bioluminescence imaging; p/s/cm^2^/sr, photons per second per square centimeter per steradian; p/s, photons per second; FACS, fluorescence-activated cell sorting; RNA-seq, RNA sequencing; GSEA, gene set enrichment analysis; NES, normalized enrichment score; IFNG, interferon gamma; GZMB, granzyme B; SEM, scanning electron microscopy; TEM, transmission electron microscopy; KEGG, Kyoto Encyclopedia of Genes and Genomes; TIGIT, T cell immunoreceptor with Ig and ITIM domains; anti-TIGIT, anti TIGIT antibody; NKG2A, natural killer group 2 member A; anti-NKG2A, anti NKG2A antibody; PD-1, programmed cell death protein 1; anti-PD-1, anti PD-1 antibody; IF, immunofluorescence; PD-L1, programmed cell death 1 ligand 1; *CD274*, programmed cell death 1 ligand 1 gene; DAPI, 4′,6-diamidino-2-phenylindole; oe, overexpression; SD, standard deviation; ANOVA, analysis of variance.

To evaluate the impact of tumor-intrinsic *NSUN2* on NK cell function, we performed in vitro cytotoxicity assays. *NSUN2* KO in MHCC-97H and Huh7 cells enhanced NK cell-mediated cytotoxicity at the tested E:T ratios (Fig. 2A and Fig. S2A and B). To recapitulate these interactions in vivo, we employed a sequential peritoneal injection model in NOD/SCID mice. This revealed that *NSUN2*-KO tumor cells were more susceptible to NK cell-mediated cytotoxicity, with consistent reductions in tumor burden observed on days 14 and 21 (Fig. 2B and Fig. S2C).

To elucidate the mechanism by which *NSUN2* conferred resistance to NK cell-mediated cytotoxicity, we cocultured NK cells with control or *NSUN2*-KO tumor cells. After fluorescence-activated cell sorting, NK cells were subjected to transcriptomic profiling (Fig. 2C). RNA-seq analysis identified 1,064 up-regulated genes and 977 down-regulated genes in NK cells cocultured with *NSUN2*-KO tumor cells compared to those cocultured with control cells (Fig. 2D). Notably, the expression of genes encoding cytokines and cytotoxic molecules was significantly elevated, including interleukin 2 receptor subunit alpha (*IL2RA*), interferon gamma (*IFNG*), granzyme B (*GZMB*), tumor necrosis factor (*TNF*), and perforin 1 (*PRF1*). Similarly, GSEA showed significant enrichment of the “natural killer cell-mediated cytotoxicity” signature in NK cells cocultured with *NSUN2*-KO cells (Fig. 2E). Consistently, flow cytometry verified increased production of IFNG and GZMB in these NK cells (Fig. 2F and Fig. S2D). As the formation of the immunological synapse and membrane protrusions are critical for NK cell-mediated killing [36,37], we utilized SEM and TEM for morphological assessment. More abundant membrane protrusions were observed on NK cells cocultured with *NSUN2*-KO tumor cells (Fig. 2G and Fig. S2E), suggesting enhanced immune synapse formation and cytotoxic activity.

Parallel RNA-seq analysis of *NSUN2*-KO and control tumor cells revealed that *NSUN2* KO up-regulated immune-related pathways (Fig. 2H). Considering that immune checkpoints facilitated tumor evasion [38], we screened for differentially expressed checkpoint genes and found that *NSUN2* deficiency led to the down-regulation of 5 key immune checkpoints (Fig. 2I). To identify the functional driver among these candidates, we treated NK cell-tumor cell cocultures with neutralizing antibodies targeting PD-1, T cell immunoreceptor with Ig and ITIM domains (TIGIT), or natural killer group 2 member A (NKG2A). Notably, only anti-PD-1 treatment effectively restored NK cell-mediated cytotoxicity (Fig. 2J) and increased the production of IFNG and GZMB by NK cells (Fig. S2F), whereas blockade of TIGIT or NKG2A had no significant effect. Guided by these functional results, we focused on the PD-L1 axis and found that *NSUN2* deficiency significantly reduced PD-L1 expression. Consistently, Western blotting, flow cytometry, and IF staining confirmed a substantial reduction in PD-L1 protein levels (Fig. 2K and Fig. S3A to C). To further establish the causal role of the *NSUN2*/PD-L1 axis, we performed reciprocal rescue experiments. In *NSUN2*-KO cells, the ectopic expression of PD-L1 (*CD274*-oe) abolished the enhanced sensitivity to NK cell-mediated cytotoxicity (Fig. S3D). Conversely, while *NSUN2* overexpression suppressed NK cell-mediated cytotoxicity, this immunosuppressive effect was effectively reversed by the concurrent knockdown of PD-L1 (Fig. S3E).

Given that the PD-1/PD-L1 axis was a canonical immune checkpoint that primarily inhibited T-cell immunity, we further investigated whether the *NSUN2*/PD-L1 axis also impacted CD8^+^ T cell function. In vivo immune depletion assays demonstrated that the anti-tumor efficacy of *Nsun2* knockout was predominantly driven by NK cells. Specifically, the suppression of tumor growth in *Nsun2*-KO models was abolished by NK cell depletion but remained largely unaffected by CD8^+^ T cell elimination (Fig. S4). These findings demonstrated that tumor-intrinsic *NSUN2* promoted resistance to NK cell-mediated cytotoxicity, at least partially, by maintaining high levels of PD-L1.

### NSUN2 modulated glycolytic metabolism and m^5^C modification of metabolic enzyme transcripts

**Fig. 3.**
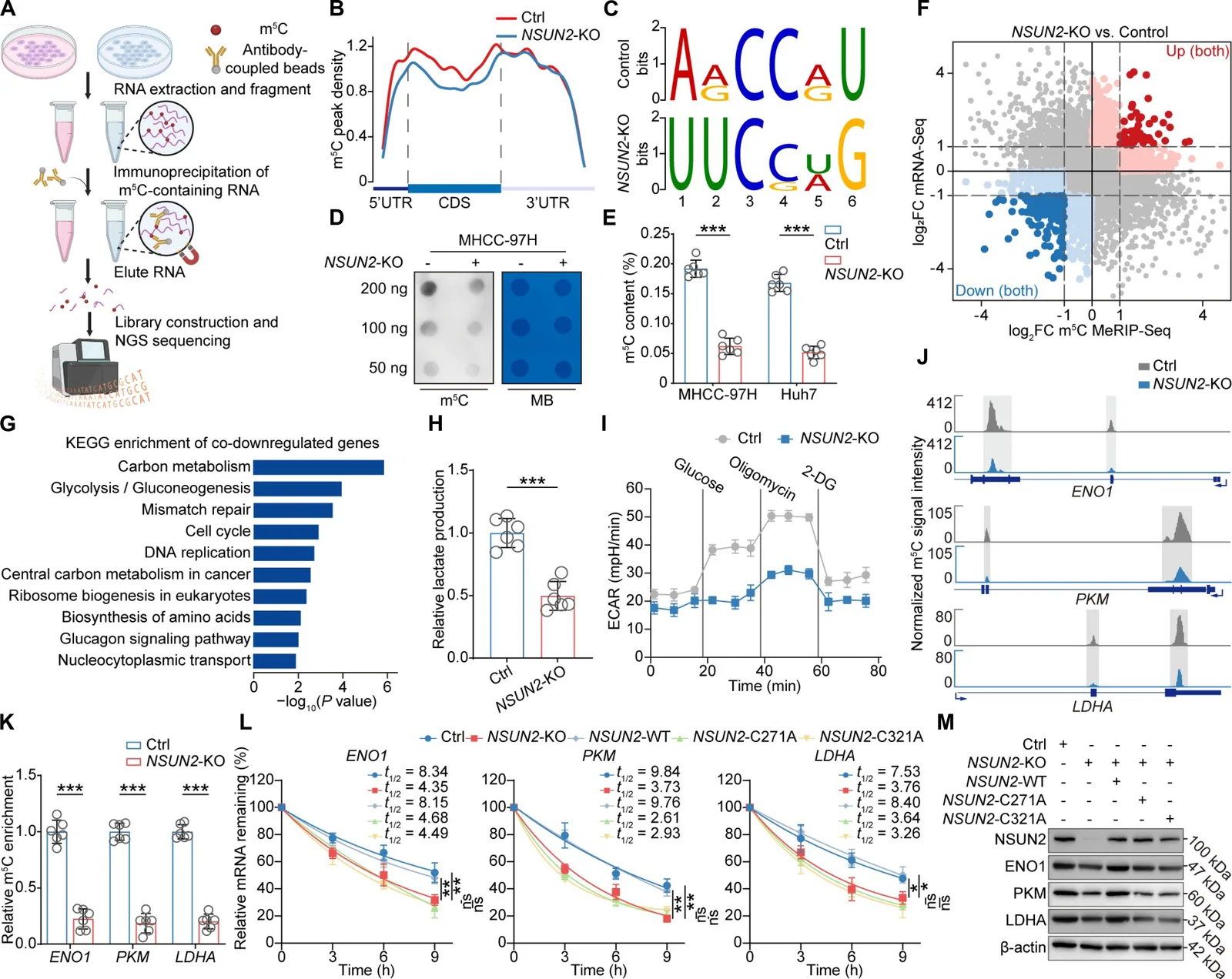
*NSUN2* modulated glycolytic metabolism and m^5^C modification of metabolic enzyme transcripts. (A) Schematic illustration of the m^5^C MeRIP-seq workflow in MHCC-97H cells. (B) Metagene distribution of m^5^C peaks along the entire transcriptome in control and *NSUN2-*KO MHCC-97H cells. (C) Motif sequence enriched in m^5^C peaks identified by MeRIP-seq in control and *NSUN2*-KO cells. (D) Dot blot assay quantifying total m^5^C abundance in control and *NSUN2-*KO MHCC-97H cells. MB staining served as a loading control. (E) Quantification of global m^5^C content in control and *NSUN2-*KO MHCC-97H cells and Huh7 cells using ELISA. (F) Four-quadrant diagram showing co-up-regulated and co-down-regulated genes of MeRIP-seq and RNA-seq. (G) KEGG pathway enrichment analysis of the co-down-regulated genes identified in (F). (H) Relative lactate production in control and *NSUN2-*KO MHCC-97H cells. (I) ECAR measured by a glycolysis stress test in control and *NSUN2-*KO MHCC-97H cells. (J) Visualization of m^5^C peak enrichment in *ENO1*, *PKM*, and *LDHA* from MeRIP-seq data. (K) Validation of m^5^C enrichment on *ENO1*, *PKM*, and *LDHA* transcripts in control and *NSUN2-*KO MHCC-97H cells by MeRIP-qPCR. (L) mRNA stability of *ENO1*, *PKM*, and *LDHA* transcripts in *NSUN2*-KO MHCC-97H cells rescued with WT or catalytic mutant (C271A and C321A) *NSUN2* vectors following treatment with actinomycin D. (M) Western blotting of ENO1, PKM, and LDHA expression in MHCC-97H cells under the indicated conditions. Data are presented as mean ± SD. Statistical significance was determined as follows: unpaired 2-tailed *t* tests were used for (E), (H), and (K); and 2-way ANOVA was used for (L). *P < 0.05; **P < 0.01; ***P < 0.001; ns, not significant. Abbreviations: *NSUN2*, NOP2/Sun RNA methyltransferase 2; m^5^C, 5-methylcytosine; MeRIP-seq, methylated RNA immunoprecipitation sequencing; RNA-seq, RNA sequencing; KO, knockout; WT, wild type; UTR, untranslated region; CDS, coding sequence; MB, methylene blue; ELISA, enzyme-linked immunosorbent assay; FC, fold change; KEGG, Kyoto Encyclopedia of Genes and Genomes; ECAR, extracellular acidification rate; ENO1, enolase 1; PKM, pyruvate kinase M1/2; LDHA, lactate dehydrogenase A; 2-DG, 2-deoxy-D-glucose; MeRIP-qPCR, methylated RNA immunoprecipitation quantitative polymerase chain reaction; mRNA, messenger RNA; *t*_1/2_, half-life; SD, standard deviation; ANOVA, analysis of variance.

As NSUN2 functioned as a methyltransferase responsible for the m^5^C modification of RNA [39], we performed m^5^C methylated RNA immunoprecipitation sequencing (MeRIP-seq) to elucidate its role in HCC cells (Fig. 3A). The global m^5^C methylation levels in *NSUN2-*KO cells were markedly diminished at the metagene level (Fig. 3B and Fig. S5A), and motif analysis identified enriched sequences in m^5^C peaks (Fig. 3C), which was further confirmed by dot blotting and ELISA-based quantification of m^5^C levels in MHCC-97H and Huh7 cells (Fig. 3D and E).

Integration analysis of RNA-seq and MeRIP-seq data identified 237 genes that were down-regulated in both mRNA expression and m^5^C methylation levels following *NSUN2* knockout (Fig. 3F). KEGG pathway analysis revealed that these NSUN2-regulated genes were significantly enriched in the carbon metabolism and glycolysis pathways (Fig. 3G). Metabolic assays demonstrated that *NSUN2* deficiency significantly attenuated glycolytic activity, as evidenced by reduced lactate production and decreased ECAR, accompanied by a compensatory increase in OCR (Fig. 3H and I and Fig. S5B). To accurately trace the metabolic fate of glucose, we performed LC-MS/MS-based U-^13^C_6_-glucose flux analysis. The results revealed a marked reduction in ^13^C labeling of key glycolytic intermediates coupled with an increase in ^13^C enrichment of tricarboxylic acid cycle intermediates in *NSUN2*-KO cells (Fig. S5C to E), indicating a metabolic shift away from aerobic glycolysis.

Screening of key glycolytic pathway genes identified *ENO1*, *PKM*, and *LDHA* as the most strongly down-regulated candidates at both the m^5^C methylation and expression levels (Fig. 3J and Fig. S5F). To validate the specific modification levels on these candidates, we performed MeRIP-qPCR, which demonstrated a significant reduction in m^5^C enrichment on *ENO1*, *PKM*, and *LDHA* transcripts in *NSUN2*-KO cells (Fig. 3K). Consequently, subsequent qPCR and Western blotting analyses confirmed that both the mRNA and protein levels of these genes were significantly reduced in *NSUN2*-KO MHCC-97H and Huh7 cells (Figs. S5G and S6A and B). Clinical relevance was further supported by the TCGA-LIHC cohort, which exhibited positive correlations between *NSUN2* expression and the expression levels of these glycolytic enzymes (Fig. S6C).

To determine the functional consequence of this modification loss, we performed RNA stability assays, which confirmed that *NSUN2* deficiency significantly shortened the half-lives of *ENO1*, *PKM*, and *LDHA* mRNAs (Fig. 3L). To confirm that this regulation depends on the methyltransferase activity of NSUN2, we performed rescue experiments using WT or catalytically inactive mutant (C271A/C321A) NSUN2. Notably, while the reintroduction of WT NSUN2 restored the expression of ENO1, PKM, and LDHA, the catalytic mutants failed to do so (Fig. 3L and M). These data collectively demonstrated that tumor-intrinsic *NSUN2* promoted glycolysis via m^5^C-dependent stabilization of metabolic enzyme transcripts.

### Histone lactylation was associated with glycolysis-driven PD-L1 up-regulation

**Fig. 4.**
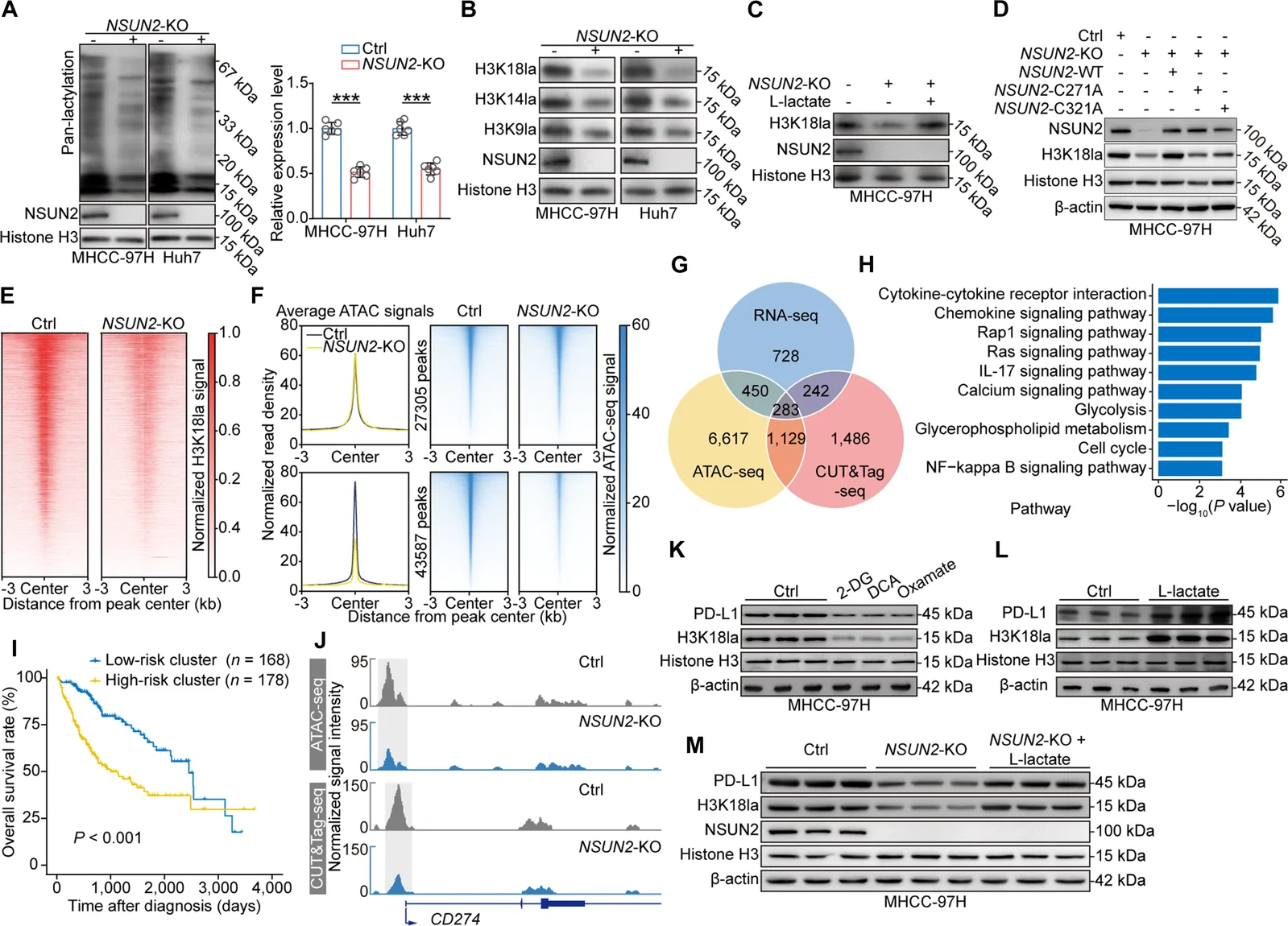
Histone lactylation was associated with glycolysis-driven PD-L1 up-regulation. (A) Western blotting (left panel) and statistical quantification (right panel) of total protein pan-lactylation levels in control and *NSUN2*-KO MHCC-97H and Huh7 cells. (B) Western blotting of site-specific histone lactylation (H3K18la, H3K14la, and H3K9la) in control and *NSUN2*-KO MHCC-97H and Huh7 cells. (C) Western blotting showing H3K18la expression in *NSUN2*-KO MHCC-97H cells treated with or without L-lactate (20 mmol/l). (D) Western blotting of H3K18la levels in *NSUN2*-KO MHCC-97H cells rescued with WT or catalytic mutant (C271A or C321A) *NSUN2*. (E) Heatmaps showing H3K18la enrichment across the genome determined by CUT&Tag-seq in control and *NSUN2*-KO MHCC-97H cells. (F) Profile plot (left panel) and heatmaps (right panel) of ATAC-seq signals reflecting chromatin accessibility in control and *NSUN2*-KO MHCC-97H cells. (G) Venn diagram showing the overlapping genes among RNA-seq, CUT&Tag-seq (H3K18la), and ATAC-seq data. (H) KEGG pathway enrichment analysis of the 283 overlapping genes identified in (G). (I) Overall survival analysis of HCC patients from the TCGA-LIHC cohort (*n* = 346) categorized into low-risk and high-risk clusters based on the 283-gene signature. (J) Visualization of ATAC-seq (top panel) and H3K18la CUT&Tag-seq (bottom panel) peaks at the *CD274* (PD-L1) promoter region in control and *NSUN2*-KO MHCC-97H cells. (K) Western blotting of PD-L1 and H3K18la expression in MHCC-97H cells treated with the glycolysis inhibitors 2-DG (10 mmol/l), DCA (20 mmol/l), or oxamate (20 mmol/l). (L and M) Western blotting showing PD-L1 and H3K18la expression in MHCC-97H cells treated with (L) exogenous L-lactate (20 mmol/l) or (M) *NSUN2*-KO cells rescued with exogenous L-lactate (20 mmol/l). Data are presented as mean ± SD. Statistical significance was determined as follows: unpaired 2-tailed *t* test was used for (A); the log-rank (Mantel–Cox) test was used for (I). *P < 0.05; **P < 0.01; ***P < 0.001; ns, not significant. Abbreviations: *NSUN2*, NOP2/Sun RNA methyltransferase 2; KO, knockout; H3K18la, histone H3 lysine 18 lactylation; H3K14la, histone H3 lysine 14 lactylation; H3K9la, histone H3 lysine 9 lactylation; ATAC-seq, assay for transposase-accessible chromatin with high-throughput sequencing; CUT&Tag-seq, cleavage under targets and tagmentation with high-throughput sequencing; kb, kilobase; bp, base pair; RNA-seq, RNA sequencing; KEGG, Kyoto Encyclopedia of Genes and Genomes; TCGA-LIHC, The Cancer Genome Atlas-Liver Hepatocellular Carcinoma; 2-DG, 2-deoxy-D-glucose; DCA, dichloroacetate; SD, standard deviation.

*NSUN2* facilitated tumor cell glycolysis, providing energy for malignant cells and generating substantial amounts of the lactate oncometabolite [40,41]. Emerging evidence indicated that lactate not only functioned as a metabolic by-product and energy substrate but also served as a lactylation donor to drive PTMs [21,22]. To investigate the nonmetabolic role of *NSUN2*-mediated glycolytic lactate production, we compared global protein lactylation levels in control and *NSUN2-*KO cells. Notably, *NSUN2* depletion significantly reduced global protein lactylation levels in both cell lines (Fig. 4A).

Examination of 3 canonical histone lactylation sites, including histone H3K18la, H3K14la, and H3K9la, revealed that *NSUN2* KO induced the most pronounced reduction at H3K18la (Fig. 4B and Fig. S7A). Furthermore, exogenous lactate supplementation effectively restored H3K18la levels in *NSUN2-*KO cells (Fig. 4C and Fig. S7B and C). Crucially, while reintroduction of WT *NSUN2* rescued this phenotype, the catalytically inactive mutant failed to restore H3K18la levels (Fig. 4D). Given that the acetyltransferase P300 was reported as a potential “writer” of histone lactylation [22], we investigated whether *NSUN2*-mediated lactylation relied on P300. Although *P300* knockdown reduced H3K18la levels, this reduction could be reversed by exogenous lactate supplementation (Fig. S7D). Moreover, *NSUN2* overexpression successfully rescued the H3K18la reduction caused by *P300* knockdown (Fig. S7E). These findings suggested that NSUN2 modulated the lactylation process primarily by regulating substrate availability (lactate) rather than being dependent on P300 levels or function.

To map the epigenetic landscape, we performed CUT&Tag-seq for H3K18la and ATAC-seq on control and *NSUN2*-KO cells. *NSUN2-*KO significantly down-regulated genome-wide H3K18la modifications (Fig. 4E and Fig. S7F and G) and reduced chromatin accessibility at 43,587 genomic loci (Fig. 4F). Integrative analysis of RNA-seq, CUT&Tag-seq, and ATAC-seq datasets identified 283 overlapping targets (Fig. 4G), which were prominently enriched in immune-related pathways (Fig. 4H). Furthermore, patients from the TCGA-LIHC cohort were stratified into high- and low-risk clusters based on the expression of the 283-gene signature, with the high-risk cohort exhibiting poorer OS (Fig. 4I).

Locus-specific analysis of the sequencing data revealed that *NSUN2* KO significantly reduced chromatin accessibility at the *CD274* (PD-L1) promoter and diminished H3K18la enrichment at the PD-L1 promoter region (Fig. 4J). Pharmacological inhibition of glycolysis using 2-DG, DCA, or oxamate down-regulated PD-L1 expression at the protein level in both MHCC-97H and Huh7 cells (Fig. 4K and Fig. S8A), whereas exogenous lactate supplementation restored its expression (Fig. 4L and Fig. S8B). In both MHCC-97H and Huh7 *NSUN2-*KO tumor cells, lactate supplementation rescued both H3K18la levels and PD-L1 expression (Fig. 4M and Fig. S8C and D). Our findings revealed that *NSUN2* drove PD-L1 expression by augmenting H3K18la levels and chromatin accessibility at the *CD274* promoter region.

### STAT3 colocalized with H3K18la and initiated transcription at the *CD274* (PD-L1) promoter region

**Fig. 5.**
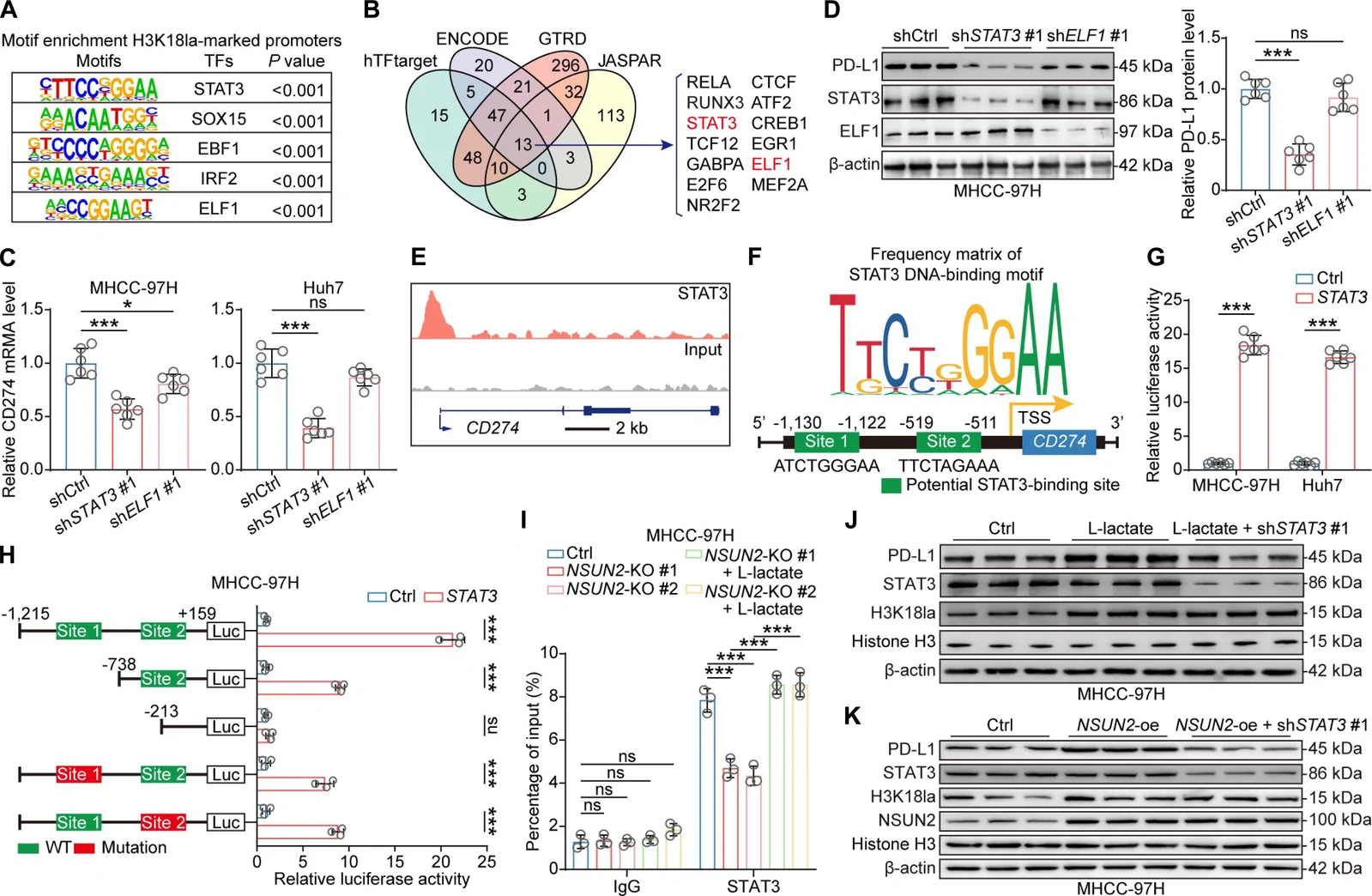
STAT3 colocalized with H3K18la and initiated transcription at the *CD274* (PD-L1) promoter region. (A) The top 5 enriched TF-binding motifs in the H3K18la-marked promoter regions of MHCC-97H cells. (B) Venn diagram indicating the overlapping TFs in the *CD274* promoter region screened using the hTFtarget, ENCODE, GTRD, and JASPAR databases. (C) Relative mRNA expression levels of *CD274* (PD-L1) in MHCC-97H and Huh7 cells after shRNA-mediated knockdown of STAT3 or ELF1. (D) Western blotting (left panel) and statistical quantification (right panel) showing PD-L1 expression following STAT3 or ELF1 knockdown in MHCC-97H cells. (E) Representative peak visualization of STAT3 binding to the *CD274* promoter detected by ChIP-seq. (F) Frequency matrix of the STAT3 DNA-binding motif from the JASPAR database (top panel) and schematic representation of 2 predicted binding sites on the *CD274* promoter upstream of the TSS (bottom panel). (G) Dual-luciferase reporter assay measuring the effect of STAT3 on *CD274* promoter activity in MHCC-97H and Huh7 cells. (H) Dual-luciferase assay evaluating the effect of STAT3 on serially truncated or mutated CD274 promoter constructs. (I) ChIP-qPCR analysis of STAT3 binding to the *CD274* promoter in control, *NSUN2*-KO, and *NSUN2*-KO MHCC-97H cells treated with L-lactate (20 mmol/l). (J and K) Western blotting showing PD-L1, STAT3, and H3K18la expression in MHCC-97H cells treated with (J) L-lactate (20 mmol/l) with or without sh*STAT3* #1, and (K) *NSUN2*-oe with or without sh*STAT3* #1. Data are presented as mean ± SD. Statistical significance was determined as follows: unpaired 2-tailed *t* tests were used for (G) and (H); 1-way ANOVA was used for (C) and (D); and 2-way ANOVA was used for (I). *P < 0.05; **P < 0.01; ***P < 0.001; ns, not significant. Abbreviations: *NSUN2*, NOP2/Sun RNA methyltransferase 2; KO, knockout; TFs, transcription factors; *CD274*, programmed cell death 1 ligand 1 gene; PD-L1, programmed cell death 1 ligand 1; STAT3, signal transducer and activator of transcription 3; ELF1, E74 like ETS transcription factor 1; shRNA, short hairpin RNA; oe, overexpression; TSS, transcription start site; kb, kilobase; ChIP-seq, chromatin immunoprecipitation sequencing; ChIP-qPCR, chromatin immunoprecipitation quantitative polymerase chain reaction; SD, standard deviation; ANOVA, analysis of variance.

To further investigate the interaction between TFs and the H3K18la-marked *CD274* promoter region, TF-binding motif analysis was performed. The analysis revealed significant enrichment of motifs for TFs, including STAT3, SRY-box transcription factor 15 (SOX15), early B-cell factor 1 (EBF1), interferon regulatory factor 2 (IRF2), and E74-like factor 1 (ELF1) at H3K18la-marked *CD274* promoter regions (Fig. 5A). Additionally, screening across 4 public databases identified 13 potential TFs, including STAT3 and ELF1 (Fig. 5B). In the TCGA-LIHC cohort, *STAT3* and *ELF1* mRNA levels positively correlated with *CD274* (PD-L1) expression (Fig. S9A). To confirm their regulatory roles in PD-L1 expression, *STAT3* or *ELF1* was knocked down in MHCC-97H and Huh7 cells. *STAT3* knockdown significantly suppressed PD-L1 expression at both mRNA and protein levels compared with *ELF1* knockdown (Fig. 5C and D and Fig. S9B).

To explore STAT3-mediated transcriptional regulation, ChIP-seq revealed robust STAT3 enrichment at the *CD274* promoter region (Fig. 5E and Fig. S9C to E). Based on the binding motif from the JASPAR database, 2 putative STAT3-binding sites were identified in the CD274 promoter (Fig. 5F). Dual-luciferase reporter assays confirmed that STAT3 significantly enhanced *CD274* promoter activity (Fig. 5G). Serial truncation and mutation analyses showed that disruption of the predicted STAT3-binding sites attenuated STAT3-induced CD274 promoter activation (Fig. 5H). To investigate the functional link between *NSUN2* and STAT3, we first assessed the impact of *NSUN2* deletion on STAT3 expression. *NSUN2-*KO did not alter total STAT3 protein levels or its phosphorylation at Y705 (Fig. S9F).

Histone lactylation played an important role in modulating chromatin architecture, transcription factor binding, and promoter accessibility [30,42]. ChIP-qPCR analysis revealed that *NSUN2-*KO cells exhibited significantly reduced STAT3 occupancy at the *CD274* promoter compared to controls. Notably, lactate supplementation restored STAT3 binding capacity in *NSUN2-*KO cells (Fig. 5I and Fig. S9G). At the protein level, *STAT3* knockdown abolished the PD-L1 up-regulation induced by lactate treatment or *NSUN2*-oe (Fig. 5J and K and Fig. S10). These results suggested that *NSUN2* promoted lactate generation, which increased H3K18la-mediated chromatin accessibility at the CD274 promoter and recruited STAT3 to the promoter to initiate transcription.

### Combination of a STAT3 inhibitor with anti-PD-L1 suppressed HCC progression in vivo

TTI-101, a small-molecule inhibitor of STAT3, has demonstrated efficacy in the treatment of HCC [43]. It is currently undergoing clinical trials, both as a monotherapy and in combination with anti-PD-L1, to assess its potential therapeutic benefits for HCC [44]. Our mechanistic studies demonstrated that STAT3 directly binds to the H3K18la-enriched *CD274* (PD-L1) promoter region within the *NSUN2*-mediated regulatory axis, establishing STAT3 as a druggable target for intercepting PD-L1-driven NK cell immune evasion in HCC.

**Fig. 6.**
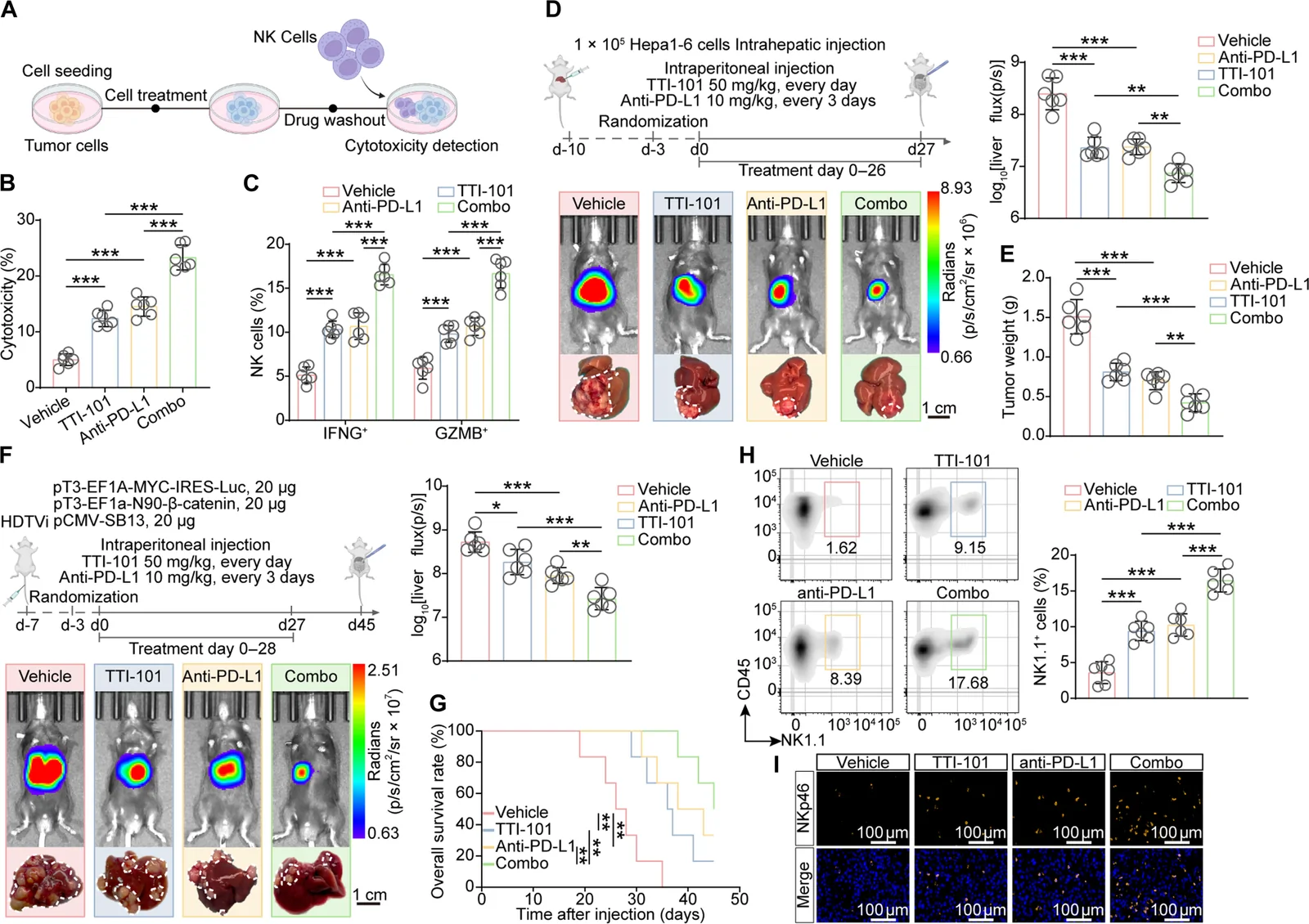
Combination of a STAT3 inhibitor with anti-PD-L1 suppressed HCC progression in vivo. (A) Schematic illustration of the experimental workflow for detecting NK cell-mediated cytotoxicity against tumor cells following the indicated treatments. (B) Quantification of NK cell-mediated cytotoxicity in MHCC-97H cells treated with vehicle, TTI-101 (STAT3 inhibitor), anti-PD-L1, or the Combo. (C) Flow cytometry analysis showing the percentages of IFNG^+^ and GZMB^+^ NK cells in the indicated groups. (D) Representative BLI and gross liver tumor images of an orthotopic HCC mouse model (injected with Hepa1-6 cells) following the indicated therapies (left panel). Statistical quantification of the bioluminescence signal is shown in the right panel. (E) Statistical quantification of the weight of dissected liver tumors from the orthotopic model in (D). (F) Representative BLI and gross liver tumor images of a spontaneous HCC mouse model under the indicated therapies (left panel). Statistical quantification of the bioluminescence signal is shown in the right panel. (G) Overall survival curves of mice in the spontaneous HCC model groups treated with the indicated therapies. (H) Representative flow cytometry plots and quantification of tumor-infiltrating NK cells (CD45^+^NK1.1^+^) in the spontaneous HCC model following the indicated treatments (left panel), with statistical quantification of NK1.1^+^ cells among CD45^+^ cells (right panel). (I) Representative IF staining of NKp46 (yellow) and DAPI (blue) in the spontaneous tumor tissues of the indicated groups. Scale bars: 100 μm. Data are presented as mean ± SD. Statistical significance was determined as follows: 1-way ANOVA was used for multiple comparisons in (B) to (F) and (H), and the log-rank (Mantel–Cox) test was used for survival analysis in (G). *P < 0.05; **P < 0.01; ***P < 0.001; ns, not significant. Abbreviations: TTI-101, STAT3 inhibitor; Combo, combination therapy; STAT3, signal transducer and activator of transcription 3; anti-PD-L1, anti-programmed cell death 1 ligand 1; NK, natural killer; HCC, hepatocellular carcinoma; IFNG, interferon gamma; GZMB, granzyme B; BLI, bioluminescence imaging; p/s/cm^2^/sr, photons per second per square centimeter per steradian; p/s, photons per second; IHC, immunohistochemistry; FACS, fluorescence-activated cell sorting; IF, immunofluorescence; NKp46, natural cytotoxicity triggering receptor 1; DAPI, 4′,6-diamidino-2-phenylindole; SD, standard deviation; ANOVA, analysis of variance.

In vitro, MHCC-97H cells were treated with vehicle, TTI-101, anti-PD-L1, or the combination (Combo), followed by coculture with NK cells isolated from human peripheral blood (Fig. 6A). Cytotoxicity assays and Annexin-V quantification concordantly revealed enhanced tumor cell death and apoptosis in the Combo therapy group compared to monotherapies or vehicle (Fig. 6B and Fig. S11A). This superior antitumor efficacy was supported by a marked enhancement of NK cell effector functions (Fig. 6C).

The therapeutic efficacy of TTI-101 combined with anti-PD-L1 was further evaluated in vivo. Orthotopic liver tumor models were established using Hepa1-6 cells, while spontaneous hepatocarcinogenesis models were generated via HDTVi of oncogenic plasmids (MYC-Luc/N90-β-catenin) and SB transposase. In both models, combination therapy reduced hepatic tumor burden, as evidenced by BLI and, in the orthotopic model, decreased tumor weight (Fig. 6D to F). Combination therapy also prolonged OS in the orthotopic and spontaneous HCC models (Fig. 6G and Fig. S11B). Posttreatment analysis of spontaneous liver tumor tissues revealed increased NK cell recruitment in the combination-treated group, as shown by flow cytometry (Fig. 6H). IF staining and subsequent quantification also demonstrated a higher density of infiltrating NKp46^+^ NK cells in spontaneous tumors (Fig. 6I and Fig. S11C). Furthermore, qPCR analysis of spontaneous tumor tissues showed that the expression of NK cell effector molecules GZMB and IFNG was markedly up-regulated following combination therapy (Fig. S11D). In summary, these findings highlighted the therapeutic value of combining TTI-101 with anti-PD-L1 therapy, with antitumor efficacy mediated by enhanced intratumoral NK cell infiltration and cytotoxicity.

### High NSUN2 expression was associated with poor prognosis and immunotherapy resistance in patients with HCC

**Fig. 7.**
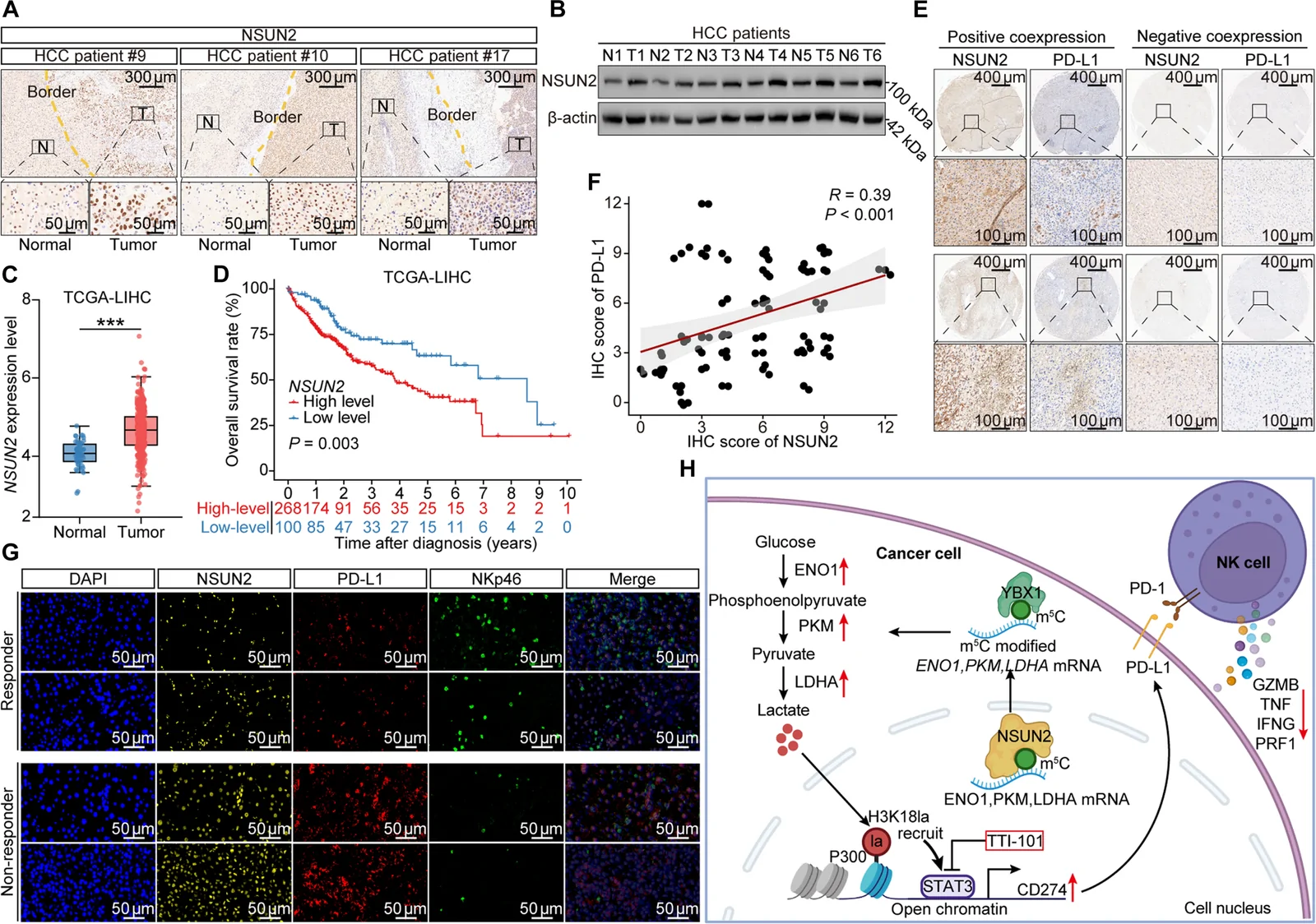
High *NSUN2* expression was associated with poor prognosis and immunotherapy resistance in patients with HCC. (A) Representative IHC images of NSUN2 expression at the interface between HCC tumor and adjacent normal tissues. Scale bars: 300 μm (top panels) and 50 μm (bottom panels). (B) Western blotting showing NSUN2 expression in paired HCC tumor and adjacent normal tissues. (C) Comparison of *NSUN2* expression in normal (*n* = 50) and tumor (*n* = 369) tissues from the TCGA-LIHC cohort. (D) Overall survival analysis of HCC patients from the TCGA-LIHC cohort (*n* = 368) categorized into high-level and low-level clusters based on *NSUN2* expression. (E) Representative IHC images of NSUN2 and PD-L1 expression in the HCC TMA. Scale bars: 400 μm (top panels) and 100 μm (bottom panels). (F) Correlation analysis between NSUN2 and PD-L1 IHC scores in the HCC TMA cohort (*R* = 0.39, *P* < 0.001). (G) Representative IF images of NSUN2 (yellow), PD-L1 (red), NKp46 (green), and DAPI (blue) in responders and nonresponders from the immunotherapy cohort. Scale bars: 50 μm. (H) Schematic model illustrating NSUN2’s role in tumor NK cell immune evasion via the m^5^C-*ENO1*/*PKM*/*LDHA* glycolytic metabolic pathway and the H3K18la-STAT3-PD-L1 transcription pathway. For IHC quantification, NSUN2 and PD-L1 staining were evaluated using IHC scores based on staining intensity and the percentage of positive tumor cells. Data are presented as mean ± SD. Statistical significance was determined as follows: an unpaired 2-tailed *t* test was used for (C); the log-rank (Mantel–Cox) test was used for survival analysis in (D); and Pearson correlation analysis was used in (F). *P < 0.05; **P < 0.01; ***P < 0.001; ns, not significant. Abbreviations: N, normal; T, tumor; *NSUN2*, NOP2/Sun RNA methyltransferase 2; HCC, hepatocellular carcinoma; IHC, immunohistochemistry; TMA, tissue microarray; STAT3, signal transducer and activator of transcription 3; TCGA-LIHC, The Cancer Genome Atlas-Liver Hepatocellular Carcinoma; IF, immunofluorescence; PD-L1, programmed cell death 1 ligand 1; NKp46, natural cytotoxicity triggering receptor 1; DAPI, 4′,6-diamidino-2-phenylindole; H3K18la, histone H3 lysine 18 lactylation; NK, natural killer; m^5^C, 5-methylcytosine; CD274, programmed cell death 1 ligand 1 gene; PD-1, programmed cell death protein 1; GZMB, granzyme B; TNF, tumor necrosis factor; IFNG, interferon gamma; PRF1, perforin 1; TTI-101, STAT3 inhibitor; YBX1, Y-box binding protein 1; ENO1, enolase 1; PKM, pyruvate kinase M1/2; LDHA, lactate dehydrogenase A; P300, E1A-binding protein p300 histone acetyltransferase; SD, standard deviation.

HCC tissues showed significantly higher NSUN2 expression than adjacent normal tissues in the snap-frozen cohort (Fig. 7A and B and Fig. S12A and B), which was confirmed by the TCGA-LIHC cohort (Fig. 7C). Elevated *NSUN2* levels were associated with poor prognosis in TCGA-LIHC and TMA cohorts (Fig. 7D and Fig. S12C). Analysis of the TCGA-LIHC cohort revealed a positive correlation between m^5^C signature expression and *CD274* (PD-L1) levels (Fig. S12D). IHC staining in the HCC TMA cohort revealed concomitant high expression of NSUN2 and PD-L1 in tumor tissues (Fig. 7E and F).

Given the crucial role of PD-L1 in ICI efficacy, we compared immunotherapy responders and nonresponders among patients with HCC using IF and IHC. Tumors from nonresponders showed higher expression levels of NSUN2, H3K18la, and PD-L1, along with decreased NK cell infiltration (Fig. 7G and Fig. S12E). Further validation in the anti-PD-1-treated HCC cohort confirmed higher *NSUN2* levels in nonresponders (Fig. S12F), suggesting that NSUN2 might contribute to immunotherapy resistance. Collectively, clinical evidence demonstrated that *NSUN2* and PD-L1 co-up-regulation was associated with poor prognosis and resistance to ICIs in HCC. Therapeutic targeting of the NSUN2/PD-L1 axis might represent a viable strategy to overcome immunotherapy resistance in HCC.

## Discussion

This study elucidated that *NSUN2*-mediated RNA m^5^C modification modulated the immunosuppressive microenvironment of HCC and identified a mechanism through which metabolic reprogramming interacted with epigenetic modifications to confer resistance to NK cell cytotoxicity (Fig. 7H). Genome-wide CRISPR screening demonstrated that *NSUN2* deficiency significantly enhanced NK cell-mediated cytotoxicity. Mechanistically, *NSUN2* facilitated lactate production via the m^5^C-dependent up-regulation of glycolytic enzymes (*ENO1*, *PKM*, and *LDHA*), increasing H3K18la-induced chromatin accessibility at the *CD274* (PD-L1) promoter region. This epigenetic remodeling promoted STAT3 recruitment and drove PD-L1 transcription. Clinically, elevated *NSUN2* expression was associated with increased PD-L1 levels, poor prognosis in patients with HCC, and higher expression in immunotherapy nonresponders, suggesting its potential as a biomarker for resistance to ICIs. Moreover, the combination of the STAT3 inhibitor TTI-101 and anti-PD-L1 therapy enhanced NK cell infiltration and cytotoxicity, suppressing tumors in vivo. These findings elucidated the molecular relationship between RNA epigenetic modifications and tumor immune evasion and proposed therapeutic strategies to overcome immunotherapy resistance in HCC.

*NSUN2*, a highly conserved RNA methyltransferase, mediates m^5^C modification in mRNA, tRNA, and rRNA [45]. RNA m^5^C plays a crucial role in tumor biology. In esophageal squamous cell carcinoma, the *NSUN2*/*YBX1* axis activated mTORC1 signaling by stabilizing *SMOX* mRNA via m^5^C-dependent mechanisms, driving ESCC progression [46]. In endometrial carcinoma, *NSUN2* conferred ferroptosis resistance via m^5^C-mediated up-regulation of *SLC7A11* [47]. Notably, *NSUN2* has been implicated in HCC and other malignancies via m^5^C-dependent stabilization of glycolytic enzyme mRNAs, enhancing glycolysis and promoting tumorigenesis [40,41]. While these studies underscore the oncogenic role of *NSUN2* in metabolism and therapy resistance, our findings advanced the field by delineating how *NSUN2*-driven glycolysis was channeled into a specific epigenetic program—histone lactylation—to directly regulate an immune checkpoint gene, thereby creating an immunosuppressive niche.

This study revealed a mechanism by which *NSUN2*-driven glycolytic reprogramming sustained tumor bioenergetics and generated lactate pools. These lactate molecules served as substrate donors for nuclear histone lactylation, linking metabolic reprogramming and epigenetic regulation in HCC. Our findings align with recent reports in acute myeloid leukemia and ovarian cancer, which similarly demonstrated that histone lactylation directly regulates PD-L1 expression [28,29]. However, our study extended this understanding by identifying NSUN2 as a novel upstream epitranscriptomic driver and STAT3 as a critical cofactor in this axis within the context of HCC. NK cells, which are crucial in innate immunity, exert potent antitumor effects by secreting cytotoxic mediators. However, immune checkpoint activation in tumor-infiltrating NK cells weakens their antitumor effector function [48–50]. The engagement of PD-1 on NK cells is associated with their subsequent functional suppression, and these cells can benefit from immunotherapy with anti-PD-1/PD-L1 antibodies [51–53]. Specifically, we found that *NSUN2*-driven metabolic reprogramming enhanced H3K18la modification, thereby increasing chromatin accessibility at the *CD274* promoter and facilitating STAT3 recruitment, which promoted PD-L1 transcription and reduced NK cell cytotoxicity. Our findings suggest that the PD-L1/PD-1 axis serves as an important downstream effector of *NSUN2* in suppressing NK cell cytotoxicity in the present HCC models, providing a mechanistic link between tumor metabolic–epigenetic remodeling and NK cell immune evasion.

Despite our findings, several limitations exist. First, while we focused on NK cell-mediated immunity, the *NSUN2*-driven metabolic shift likely influences other immune subsets, such as T cells and macrophages, necessitating broader immunological profiling. Second, because our principal in vivo immune conclusions were largely based on the Hepa1-6 model, which has relatively low immunogenicity and NK-cell dependence, the relative contribution of NK cells versus CD8^+^ T cells may not fully reflect the immune heterogeneity of human HCC. Third, although we identified glycolytic enzymes as key *NSUN2* targets, we cannot exclude other oncogenic substrates contributing to this phenotype. Fourth, our in vivo models exclusively utilized male mice to minimize hormonal variability. Given the sexual dimorphism in HCC, future validation in female models is essential to generalize these findings. Finally, our clinical observations rely on retrospective cohorts. Large-scale, prospective, multicenter studies are required to firmly establish *NSUN2* as a biomarker and assess the clinical efficacy of combining TTI-101 with anti-PD-L1 therapy.

## Conclusions

Collectively, these findings identified *NSUN2* as an important regulator of HCC immune evasion by integrating RNA methylation, metabolic reprogramming, and histone PTMs. Pharmacological targeting of *NSUN2* or its downstream pathways restored NK cell-mediated antitumor cytotoxicity, providing a rationale for combinatorial therapeutic strategies to overcome immunotherapy resistance in HCC. Notably, the combination of the STAT3 inhibitor TTI-101 and anti-PD-L1 therapy showed antitumor effects in preclinical murine models. Consequently, targeting the *NSUN2*-glycolysis-H3K18la/STAT3 axis may represent a potential strategy to enhance immunotherapy responsiveness in HCC.

## Ethical Approval

This study, including all human and animal experiments, was conducted in accordance with relevant ethical standards. The human research protocol was approved by the Institutional Research Ethics Committee of Zhongshan Hospital, Fudan University (Approval No. 2021BAT4859) and conducted in accordance with the principles of the Declaration of Helsinki. Informed consent was obtained from all patients prior to their enrollment in the study. All animal procedures were approved by the Shanghai Medical Experimental Animal Care Committee (Approval No. 20220120-068) and were performed in accordance with the guidelines of the National Academy of Sciences and the National Institutes of Health.

## Data Availability

The RNA-seq, ATAC-seq, CUT&Tag-seq, MeRIP-seq, and ChIP-seq data generated in this study have been deposited in the National Omics Data Encyclopedia (https://www.biosino.org/node) [54] under accession numbers OEP00006779 and OEP00006780. Additional data supporting the findings of this study are available from the corresponding authors upon reasonable request.
