# Supplementary material for: Metabolic Reprogramming of NSUN2-Mediated m5C Modification Promotes the Progression of Hepatocellular Carcinoma by Restricting Natural Killer Cell-Mediated Cytotoxicity
Source: Cancer Commun (Lond). 2026 Aug 31;46:0043. doi: 10.34133/cancomm.0043 (PMC13527209; doi:10.34133/cancomm.0043)
Supplement: Supplementary file 1 [file cancomm.0043.f1.docx]

**Supplementary Materials**

**Metabolic reprogramming of *NSUN2*-mediated m^5^C modification promotes the progression of hepatocellular carcinoma by restricting natural killer cell-mediated cytotoxicity**

**Contents**

Supplementary Figures S1-S12

Supplementary Tables S1-S7

**Supplementary Figures**

**Supplementary Figure S1. *NSUN2* expression was negatively associated with NK cell infiltration and effector function in HCC.** (A) Heatmap illustrating the expression of lineage-specific marker genes across major cell types identified in the GSE156625, GSE202642, and GSE149614 datasets. (B) Box plot comparing NK cell percentages within the immune cell populations of HCC and adjacent normal tissues (*n* = 26 per group). (C) Violin plots comparing the expression of NK cell-mediated cytotoxicity genes (*PRF1*, *IFNG*, *GZMA*, and *GZMB*) between HCC and adjacent normal tissues. (D-E) Representative images of H&E, Ki-67, and NKp46 IHC staining in tumor tissues and paired adjacent normal tissues from patients with HCC. (F) Quantification of NKp46^+^ cells per HPF. Related to Figure 1A. (G) Representative gating strategy of CD45^+^ cells in HCC patient tissues. Related to Figure 1B. (H) Proportion of IFNG^+^ and GZMB^+^ NK cells in HCC and adjacent normal tissues, as detected by flow cytometry. Related to Figure 1B. (I) Scatter plot showing the correlation between *NSUN2* expression and the expression of NK signature genes in the TCGA-LIHC cohort (*R* = -0.33, *P* < 0.001). (J) Tumor weight of control and *Nsun2*-KO subcutaneous tumors. Related to Figure 1G. (K) Quantification of NK1.1^+^ cells per HPF in control and *Nsun2*-KO subcutaneous tumors. Related to Figure 1H. For IF quantification, marker-positive cells were counted in randomly selected HPFs. Data are presented as mean ± SD. Statistical significance was determined as follows: unpaired two-tailed Student's *t*-tests were used for (B), (C), (F), (H), (J), and (K); and Pearson's correlation analysis was used for (I). * *P* < 0.05; ** *P* < 0.01; *** *P* < 0.001; ns, not significant.

Abbreviations: *NSUN2*, NOP2/Sun RNA methyltransferase 2; NK, natural killer; DC, dendritic cell; HCC, hepatocellular carcinoma; *PRF1*, perforin 1; *IFNG*, interferon gamma; *GZMA*, granzyme A; *GZMB*, granzyme B; N, normal; T, tumor; H&E, hematoxylin and eosin; NKp46, natural cytotoxicity triggering receptor 1; Ki-67, marker of proliferation Ki-67; HPF, high-power field; SSC-A, side scatter area; APC-Cy7, allophycocyanin-cyanine7; BV510, brilliant violet 510; TCGA-LIHC, The Cancer Genome Atlas-Liver Hepatocellular Carcinoma; SD, standard deviation; R, correlation coefficient.

**Supplementary Figure S2. Validation of *NSUN2* knockout efficiency and its effect on NK cell cytotoxicity.** (A) Western blotting confirming the knockout efficiency of NSUN2 in MHCC-97H (top panel) and Huh7 (bottom panel) cells. (B) NK cell-mediated cytotoxicity against control and *NSUN2*-KO Huh7 cells at the indicated E:T ratios. (C) Statistical quantification of bioluminescence intensity from the *in vivo* cytotoxicity assay in NOD/SCID mice at the indicated time points. Related to Figure 2B. (D) Statistical analysis of the percentages of IFNG^+^ and GZMB^+^ NK cells following co-culture with control or *NSUN2*-KO tumor cells. Related to Figure 2F. (E) Quantification of the mean number of membrane protrusions per cell on NK cells co-cultured with control or *NSUN2*-KO tumor cells based on SEM and TEM imaging. Related to Figure 2G. (F) Statistical analysis of IFNG^+^ (left panel) and GZMB^+^ (right panel) NK cell percentages after co-culture with tumor cells under the indicated treatment conditions. Data are presented as mean ± SD. Statistical significance was determined as follows: unpaired two-tailed Student's *t*-tests were used for (D) and (E); one-way ANOVA was used for (F); and two-way ANOVA was used for (B) and (C). * *P* < 0.05; ** *P* < 0.01; *** *P* < 0.001; ns, not significant.

Abbreviations: *NSUN2*, NOP2/Sun RNA methyltransferase 2; KO, knockout; E:T, effector-to-target; NOD/SCID, nonobese diabetic/severe combined immunodeficiency; BLI, bioluminescence imaging; p/s, photons per second; *IFNG*, interferon gamma; *GZMB*, granzyme B; SEM, scanning electron microscopy; TEM, transmission electron microscopy; oe, overexpression; TIGIT, T cell immunoreceptor with Ig and ITIM domains; NKG2A, natural killer group 2 member A; PD-1, programmed cell death protein 1; SD, standard deviation; ANOVA, analysis of variance.

**Supplementary Figure S3. *NSUN2*-regulated PD-L1 expression to modulate HCC cell sensitivity to NK cell-mediated cytotoxicity.** (A) Statistical quantification of the relative PD-L1 fluorescence intensity in control and *NSUN2*-KO MHCC-97H cells. Related to Figure 2K. (B) Western blotting of NSUN2 and PD-L1 protein expression levels in control and two *NSUN2*-KO MHCC-97H clones (left panel), with statistical quantification of relative PD-L1 protein level (right panel). (C) Representative flow cytometry histograms (left panel) and statistical quantification of the percentage of PD-L1^+^ cells in control and *NSUN2*-KO MHCC-97H cells (right panel). (D) Western blotting of NSUN2 and PD-L1 expression in the indicated groups (left panel). NK cell-mediated cytotoxicity assay showing that *CD274*-oe reversed the sensitization of *NSUN2*-KO tumor cells to NK cells (right panel). (E) Western blotting of NSUN2 and PD-L1 expression in MHCC-97H cells under the indicated treatment conditions (left panel). NK cell-mediated cytotoxicity assay showing that PD-L1 knockdown (sh*CD274*) partially reversed the reduction in tumor cell sensitivity to NK cells induced by *NSUN2* overexpression (right panel). PD-L1 fluorescence intensity was quantified using ImageJ software. Data are presented as mean ± SD. Statistical significance was determined as follows: An unpaired two-tailed Student's *t*-test was used for (A); one-way ANOVA was used for (B) and (C), and two-way ANOVA was used for (D) and (E). * *P* < 0.05; ** *P* < 0.01; *** *P* < 0.001; ns, not significant.

Abbreviations: *NSUN2*, NOP2/Sun RNA methyltransferase 2; KO, knockout; oe, overexpression; PD-L1, programmed cell death 1 ligand 1; *CD274*, programmed cell death 1 ligand 1 gene; shRNA, short hairpin RNA; SD, standard deviation; ANOVA, analysis of variance.

**Supplementary Figure S4. *Nsun2* deficiency suppressed tumor progression through an NK cell-dependent but CD8^+^ T cell-independent mechanism.** (A) Gross images of subcutaneous tumors in the indicated groups (left panel) and statistical quantification of tumor weight (right panel, *n* = 5 per group). Scale bar: 1 cm. (B) Representative IF images of CD8^+^ cells (red) and DAPI (blue) in subcutaneous tumors from the indicated groups, verifying the efficiency of CD8^+^ T cell depletion. Scale bars: 200 µm. (C) Representative BLI images of orthotopic tumors at day 7 and day 21 post-injection (left panel), and statistical quantification of bioluminescence intensity (right panel, *n* = 6 per group). Data are presented as mean ± SD. Statistical significance was determined as follows: one-way ANOVA was used for (A) and (C). * *P* < 0.05; ** *P* < 0.01; *** *P* < 0.001; ns, not significant.

Abbreviations: *NSUN2*, NOP2/Sun RNA methyltransferase 2; KO, knockout; anti-NK, anti-NK1.1 antibody; anti-CD8, anti-CD8 antibody; IF, immunofluorescence; DAPI, 4',6-diamidino-2-phenylindole; BLI, bioluminescence imaging; p/sec/cm^2^/sr, photons per second per square centimeter per steradian; p/s, photons per second; SD, standard deviation; ANOVA, analysis of variance.

**Supplementary Figure S5. *NSUN2* promoted glycolysis through m^5^C** **modification of metabolic enzyme transcripts.** (A) Pie charts showing the distribution of m^5^C methylation sites in different transcript regions in control and *NSUN2*-KO MHCC-97H cells. (B) OCR profiles of control and *NSUN2*-KO MHCC-97H cells. (C) Schematic illustration of metabolic profiles in the TCA cycle. (D-E) LC/MS-based metabolic flux analysis using U-^13^C_6_-glucose labeling in control and *NSUN2*-KO MHCC-97H cells. Quantification of the relative abundance of (D) key glycolytic/lactate intermediates and (E) TCA cycle intermediates. (F) Comprehensive glycolysis flow chart (left panel) and heatmap showing the differential expression profiles of key glycolytic enzymes in control and *NSUN2*-KO cells derived from RNA-seq data (right panel). (G) Relative mRNA expression levels of *ENO1*, *PKM*, and *LDHA* in MHCC-97H and Huh7 cells. Data are presented as mean ± SD. Statistical significance was determined as follows: unpaired two-tailed Student's *t*-tests were used for (D), (E), and (G). * *P* < 0.05; ** *P* < 0.01; *** *P* < 0.001; ns, not significant.

Abbreviations: NSUN2, NOP2/Sun RNA methyltransferase 2; KO, knockout; CDS, coding sequence; UTR, untranslated region; OCR, oxygen consumption rate; FCCP, carbonyl cyanide 4-(trifluoromethoxy) phenylhydrazone; TCA, tricarboxylic acid; G-6-P, glucose-6-phosphate; F-6-P, fructose-6-phosphate; F-1,6-BP, fructose-1,6-bisphosphate; G-3-P, glyceraldehyde-3-phosphate; 1,3-BPG, 1,3-bisphosphoglycerate; 3-PG, 3-phosphoglycerate; 2-PG, 2-phosphoglycerate; PEP, phosphoenolpyruvate; Pyr, pyruvate; Lac, lactate; Cit, citrate; IsoCit, isocitrate; α-KG, alpha-ketoglutarate; Suc, succinate; Fum, fumarate; Mal, malate; OAA, oxaloacetate; *SLC2A1*, solute carrier family 2 member 1; *SLC2A3*, solute carrier family 2 member 3; *HK1*, hexokinase 1; *HK2*, hexokinase 2; *GPI*, glucose-6-phosphate isomerase; *PFKL*, phosphofructokinase, liver type; *PFKM*, phosphofructokinase, muscle type; *PFKP*, phosphofructokinase, platelet type; *ALDOA*, aldolase, fructose-bisphosphate A; *ALDOC*, aldolase, fructose-bisphosphate C; *TPI1*, triosephosphate isomerase 1; *GAPDH*, glyceraldehyde-3-phosphate dehydrogenase; *PGK1*, phosphoglycerate kinase 1; *PGAM1*, phosphoglycerate mutase 1; *ENO1*, enolase 1; *ENO2*, enolase 2; *PKM*, pyruvate kinase M1/2; *LDHA*, lactate dehydrogenase A; *LDHB*, lactate dehydrogenase B; mRNA, messenger RNA; SD, standard deviation.

**Supplementary Figure S6. *NSUN2* expression was positively correlated with glycolytic enzyme expression and maintained their protein levels.** (A-B) Western blotting (left panel) and statistical quantification (right panel) of ENO1, PKM, and LDHA expression in control and *NSUN2*-KO (A) MHCC-97H and (B) Huh7 cells. (C) Correlation analysis between *NSUN2* and *PKM* (*R* = 0.33, *P* < 0.001), *ENO1* (*R* = 0.36, *P* < 0.001) *and LDHA* (*R* = 0.32, *P* < 0.001) expression levels in the TCGA-LIHC cohort. Data are presented as mean ± SD. Statistical significance was determined as follows: one-way ANOVA was used for (A) and (B); and Pearson's correlation analysis was used for (C). * *P* < 0.05; ** *P* < 0.01; *** *P* < 0.001; ns, not significant.

Abbreviations: NSUN2, NOP2/Sun RNA methyltransferase 2; KO, knockout; *ENO1*, enolase 1; *PKM*, pyruvate kinase M1/2; LDHA, lactate dehydrogenase A; TCGA-LIHC, The Cancer Genome Atlas-Liver Hepatocellular Carcinoma; TPM, transcripts per million; R, correlation coefficient; SD, standard deviation; ANOVA, analysis of variance.

**Supplementary Figure S7. *NSUN2*-mediated glycolysis promoted PD-L1 expression via H3K18 lactylation.** (A) Statistical quantification of site-specific histone lactylation levels (H3K18la, H3K14la, and H3K9la) in control and *NSUN2*-KO MHCC-97H and Huh7 cells. Related to Figure 4B. (B-C) Statistical quantification (B) and representative Western blotting with quantification (C) of H3K18la levels in control and *NSUN2*-KO MHCC-97H and Huh7 cells treated with or without L-lactate (20 mmol/L). Related to Figure 4C. (D-E) Western blotting and statistical quantification of P300 and H3K18la levels in cells treated with L-lactate (20 mmol/L) or *NSUN2* overexpression combined with *P300* knockdown. (F-G) Genomic feature distribution (F) and metagene profile plot (G) of H3K18la CUT&Tag signals in control and *NSUN2*-KO MHCC-97H cells. Related to Figure 4E. Data are presented as mean ± SD. Statistical significance was determined as follows: unpaired two-tailed Student's *t*-test was used for (A); one-way ANOVA was used for (B) and (C); and two-way ANOVA was used for (D) and (E). * *P* < 0.05; ** *P* < 0.01; *** *P* < 0.001; ns, not significant.

Abbreviations: NSUN2, NOP2/Sun RNA methyltransferase 2; KO, knockout; siRNA, small interfering RNA; P300, E1A-binding protein p300 histone acetyltransferase; H3K18la, histone H3 lysine 18 lactylation; H3K14la, histone H3 lysine 14 lactylation; H3K9la, histone H3 lysine 9 lactylation; UTR, untranslated region; kb, kilobase; SD, standard deviation; ANOVA, analysis of variance.

**Supplementary Figure S8. H3K18 lactylation mediated glycolysis-driven PD-L1 upregulation.** (A) Western blotting and statistical quantification of PD-L1 and H3K18la expression in Huh7 cells treated with the glycolysis inhibitors 2-DG (10 mmol/L), DCA (20 mmol/L), or Oxamate (20 mmol/L). (B-C) Western blotting and statistical quantification showing PD-L1 and H3K18la expression in Huh7 cells treated with (B) exogenous L-lactate (20 mmol/L) or (C) NSUN2-KO cells rescued with exogenous L-lactate (20 mmol/L). (D) Statistical quantification of relative PD-L1 protein levels in MHCC-97H cells treated with glycolysis inhibitors, L-lactate (20 mmol/L), or *NSUN2*-KO rescue with L-lactate (20 mmol/L). Related to Figure 4K-M. Data are presented as mean ± SD. Statistical significance was determined as follows: unpaired two-tailed Student's *t*-tests were used for (B) and (D, middle panel); one-way ANOVA was used for (A), (C), and (D, left and right panels). * *P* < 0.05; ** *P* < 0.01; *** *P* < 0.001; ns, not significant.

Abbreviations: NSUN2, NOP2/Sun RNA methyltransferase 2; KO, knockout; H3K18la, histone H3 lysine 18 lactylation; PD-L1, programmed cell death 1 ligand 1; 2-DG, 2-deoxy-D-glucose; DCA, dichloroacetate; SD, standard deviation; ANOVA, analysis of variance.

**Supplementary Figure S9. STAT3 colocalized with H3K18la at the *CD274* (PD-L1) promoter region and initiated transcription.** (A) Pearson correlation analysis between *STAT3* and *CD274* (*R* = 0.35, *P* < 0.001) (left panel) and *ELF1* and *CD274* (*R* = 0.34, *P* < 0.001) (right panel) using the TCGA-LIHC cohort. (B) Western blotting (left panel) and statistical quantification (right panel) of PD-L1 expression in Huh7 cells following shRNA-mediated knockdown of *STAT3* or *ELF1* (sh*STAT3* #1 or sh*ELF1* #1). (C-D) Profile plot (C) and heatmap (D) of STAT3 ChIP-seq signals relative to the input in the TSS region. (E) De novo motif of STAT3 identified from ChIP-seq analysis. (F) Western blotting (left panel) and statistical quantification (right panel) showing the expression of NSUN2, pSTAT3 (Y705), and total STAT3 under the indicated conditions (control, *NSUN2*-KO, *NSUN2*-oe, or L-lactate treatment) in MHCC-97H cells. (G) ChIP-qPCR analysis of the binding of STAT3 to the *CD274* promoter in control and *NSUN2*-KO Huh7 cells treated with or without L-lactate (20 mmol/L). Data are presented as mean ± SD. Statistical significance was determined as follows: one-way ANOVA was used for (B) and (F); two-way ANOVA was used for (G); and Pearson's correlation analysis was used for (A). * *P* < 0.05; ** *P* < 0.01; *** *P* < 0.001; ns, not significant.

Abbreviations: TCGA-LIHC, The Cancer Genome Atlas-Liver Hepatocellular Carcinoma; TPM, transcripts per million; *CD274*, programmed cell death 1 ligand 1 gene; PD-L1, programmed cell death 1 ligand 1 protein; *STAT3*, signal transducer and activator of transcription 3; *ELF1*, E74-like ETS transcription factor 1; sh*STAT3*, shRNA targeting *STAT3*; sh*ELF1*, shRNA targeting *ELF1*; shCtrl, control short hairpin RNA; TSS, transcription start site; kb, kilobase; *NSUN2*, NOP2/Sun RNA methyltransferase 2; KO, knockout; oe, overexpression; pSTAT3 (Y705), STAT3 phosphorylated at tyrosine 705; IgG, immunoglobulin G; ChIP-qPCR, chromatin immunoprecipitation-quantitative polymerase chain reaction; SD, standard deviation; ANOVA, analysis of variance.

**Supplementary Figure S10. STAT3 was required for lactate-induced or *NSUN2*-mediated PD-L1 expression.** (A) Statistical quantification of relative PD-L1 protein levels in MHCC-97H cells under the indicated conditions. Related to Figure 5J-K. (B-C) Western blotting (left panel) and statistical quantification (right panel) showing the expression of PD-L1, STAT3, and H3K18la in Huh7 cells treated with (B) L-lactate (20 mmol/L) with or without sh*STAT3* #1, and (C) *NSUN2*-oe with or without sh*STAT3* #1. Data are presented as mean ± SD. Statistical significance was determined as follows: one-way ANOVA was used for (A), (B), and (C). * *P* < 0.05; ** *P* < 0.01; *** *P* < 0.001; ns, not significant.

Abbreviations: NSUN2, NOP2/Sun RNA methyltransferase 2; PD-L1, programmed cell death 1 ligand 1; *STAT3*, signal transducer and activator of transcription 3; H3K18la, histone H3 lysine 18 lactylation; oe, overexpression; sh*STAT3*, shRNA targeting *STAT3*; SD, standard deviation; ANOVA, analysis of variance.

**Supplementary Figure S11. Combination of a STAT3 inhibitor with anti-PD-L1 promoted NK cell-mediated cytotoxicity and thereby inhibited HCC progression.** (A) Representative flow cytometry histograms (left) and statistical quantification (right) of NK cell-mediated cytotoxicity (indicated by Annexin-V^+^ tumor cells) in NK cell-tumor cell co-culture systems treated with Vehicle, TTI-101, anti-PD-L1, or Combo. (B) Overall survival curves of mice in the orthotopic HCC model groups treated with the indicated therapies. Related to Figure 6D. (C) Statistical quantification of NKp46^+^ cells per HPF in spontaneous HCC tumor tissues. Related to Figure 6I. (D) Fold change in mRNA levels of *Gzmb* and *Ifng* in tumor tissues from the spontaneous HCC model. For IF quantification, NKp46-positive cells were counted in randomly selected HPFs. Data are presented as mean ± SD. Statistical significance was determined as follows: one-way ANOVA was used for (A), (C), and (D); and the log-rank (Mantel-Cox) test was used for survival analysis in (B). * *P* < 0.05; ** *P* < 0.01; *** *P* < 0.001; ns, not significant.

Abbreviations: Combo, combination therapy; anti-PD-L1, anti-programmed cell death 1 ligand 1 antibody; TTI-101, STAT3 inhibitor; NK, natural killer; HCC, hepatocellular carcinoma; Gzmb, granzyme B; Ifng, interferon gamma; HPF, high-power field; FITC, fluorescein isothiocyanate; SD, standard deviation; ANOVA, analysis of variance.

**Supplementary Figure S12. NSUN2 was overexpressed in HCC and associated with immunotherapy resistance.** (A) Representative IHC staining images of NSUN2 in paired HCC tumor and adjacent normal tissues (left panel), and quantitative comparison of the IHC scores between these paired samples (right panel). Scale bars: 100 µm. (B) Relative protein expression levels of NSUN2 in paired adjacent normal and HCC tumor tissues. Related to Figure 7B. (C) Overall survival analysis of HCC patients in the TMA cohort divided into high and low NSUN2 expression groups based on IHC staining. (D) Correlation analysis between m^5^C signature expression and *CD274* expression in the TCGA-LIHC dataset (*R* = 0.33, *P* < 0.001). (E) Comparison of NSUN2, PD-L1 and H3K18la expression between responders (*n* = 15) and non-responders (*n* = 15) from the immunotherapy cohort. (F) Statistical quantification of *NSUN2* expression levels in immunotherapy responders (*n* = 6) and non-responders (*n* = 29) in the ERP117672 cohort. Data are presented as mean ± SD. Statistical significance was determined as follows: paired two-tailed Student's t-tests were used for (A) and (B); unpaired two-tailed Student's *t*-tests were used for (E) and (F); the log-rank test was used for (C); and Pearson's correlation analysis was used for (D). * *P* < 0.05; ** *P* < 0.01; *** *P* < 0.001; ns, not significant.

Abbreviations: NSUN2, NOP2/Sun RNA methyltransferase 2; IHC, immunohistochemistry; HCC, hepatocellular carcinoma; TCGA-LIHC, The Cancer Genome Atlas-Liver Hepatocellular Carcinoma; CD274, programmed cell death 1 ligand 1 gene; PD-L1, programmed cell death 1 ligand 1; H3K18la, histone H3 lysine 18 lactylation; R, correlation coefficient; m^5^C, 5-methylcytosine; SD, standard deviation.

**Supplementary Tables**

**Supplementary Table S1. Clinical characteristics of HCC patients (*n* = 415).**

|  | | **Snap-frozen cohort**  **(*n* = 30)** | **Immunotherapy cohort**  **(*n* = 30)** | **Tissue microarray**  **cohort**  **(*n* = 355)** |
| --- | --- | --- | --- | --- |
| **Clinical characteristics** | | | | |
| Sex | Male | 18 | 21 | 196 |
|  | Female | 12 | 9 | 159 |
| Age (years) | >50 | 21 | 19 | 187 |
|  | ≤50 | 9 | 11 | 168 |
| Liver cirrhosis | Yes | 19 | 14 | 205 |
|  | No | 11 | 16 | 150 |
| ALT (U/L) | ≤40 | 14 | 13 | 189 |
|  | >40 | 16 | 17 | 166 |
| AST (U/L) | ≤40 | 16 | 12 | 191 |
|  | >40 | 14 | 18 | 164 |
| γ-GT (U/L) | ≤45 | 12 | 16 | 164 |
|  | >45 | 18 | 14 | 191 |
| CEA (ng/mL) | ≤5 | 28 | 23 | 310 |
|  | >5 | 2 | 7 | 45 |
| AFP (ng/mL) | ≤400 | 14 | 12 | 126 |
|  | >400 | 16 | 18 | 229 |
| Tumor number | Single | 23 | 10 | 238 |
|  | Multiple | 7 | 20 | 117 |
| Tumor size (cm) | ≤5 | 12 | 12 | 132 |
|  | >5 | 18 | 18 | 223 |
| Satellite lesion | No | 17 | 14 | 192 |
|  | Yes | 13 | 16 | 163 |
| PVTT | No | 27 | 21 | 253 |
|  | Yes | 3 | 9 | 102 |
| MVI | No | 24 | 12 | 231 |
|  | Yes | 6 | 18 | 124 |
| BCLC stage | 0+A | 22 | 19 | 251 |
|  | B+C | 8 | 11 | 104 |
| **Etiology** | | | | |
| Categorization | HBV-HBsAg^+^ | 20 | 19 | 241 |
|  | HBV-OBI | 2 | 0 | 12 |
|  | HCV | 1 | 2 | 15 |
|  | MASLD | 2 | 1 | 31 |
|  | MetALD | 5 | 6 | 44 |
|  | Others | 0 | 2 | 12 |

**Abbreviations**: ALT, alanine aminotransferase; AST, aspartate transaminase; AFP, α-fetoprotein; CEA, carcinoembryonic antigen; γ-GT, γ-glutamyl transferase; PVTT, portal vein tumor thrombus; MVI, microvascular invasion; BCLC, Barcelona Clinic Liver Cancer; HBV, hepatitis B virus; HBsAg, hepatitis B surface antigen; OBI, occult hepatitis B virus infection; HCV, hepatitis C virus; MASLD, metabolic dysfunction-associated steatotic liver disease; MetALD, MASLD with increased alcohol intake.

**Supplementary Table S2. Sequences of primers used for real-time quantitative PCR.**

| **Gene** | **Species** | **Forward primer** | **Reverse primer** |
| --- | --- | --- | --- |
| *NSUN2* | Human | CAAGCTGTTCGAGCACTACTAC | CTCCCTGAGAGCGTCCATGA |
| *HLA-E* | Human | TTCCGAGTGAATCTGCGGAC | GTCGTAGGCGAACTGTTCATAC |
| *HMGB1* | Human | TATGGCAAAAGCGGACAAGG | CTTCGCAACATCACCAATGGA |
| *CD274* | Human | TGGCATTTGCTGAACGCATTT | TGCAGCCAGGTCTAATTGTTTT |
| *NECTIN3* | Human | GCAGTTCACCATCCCCAATATG | TCCAAGCGGGAATGTAACAGC |
| *HLA-C* | Human | CCATGAGGTATTTGTGGACCG | TCTCGGACTCTCGTCGTCG |
| *PKM* | Human | ATAACGCCTACATGGAAAAGTG | TAAGCCCATCATCCACGTAGA |
| *ENO1* | Human | AAAGCTGGTGCCGTTGAGAA | GGTTGTGGTAAACCTCTGCTC |
| *LDHA* | Human | TTGACCTACGTGGCTTGGAAG | GGTAACGGAATCGGGCTGAAT |
| *GZMB* | Human | CCCTGGGAAAACACTCACACA | GCACAACTCAATGGTACTGTCG |
| *IFNG* | Human | TCGGTAACTGACTTGAATGTCC | TCGCTTCCCTGTTTTAGCTGC |
| *IL12A* | Human | TGCCTTCACCACTCCCAAAACC | CAATCTCTTCAGAAGTGCAAGGG |
| *Gzmb* | Mouse | CCACTCTCGACCCTACATGG | GGCCCCCAAAGTGACATTTATT |
| *Ifng* | Mouse | ATGAACGCTACACACTGCATC | CCATCCTTTTGCCAGTTCCTC |

**Supplementary Table S3. Sequences of small interfering RNAs (siRNAs) and short hairpin RNAs (shRNAs).**

| **Name** | **Sequence (5'-3')** |
| --- | --- |
| siCtrl | UUCUCCGAACGUGUCACGUAA |
| si*PUS7* #1-1 | UUUUUUGUCUCUUCAACUGGG |
| si*PUS7* #1-2 | CAGUUGAAGAGACAAAAAAAC |
| si*SETDB1* #1-1 | GGUGCAACAUGGACUACAAGU |
| si*SETDB1* #1-2 | UUGUAGUCCAUGUUGCACCAA |
| si*BCL7A* #1-1 | GGUGUGUUGUACAGCAGUAUA |
| si*BCL7A* #1-2 | UACUGCUGUACAACACACCAA |
| si*FNBP1L* #1-1 | GCGAGAAGUUGUAGCAGAAGA |
| si*FNBP1L* #1-2 | UUCUGCUACAACUUCUCGCUG |
| si*NSUN2* #1-1 | GGAAAGAGAUCCUCUUCUAUG |
| si*NSUN2* #1-2 | UAGAAGAGGAUCUCUUUCCUG |
| si*PAIP1* #1-1 | GAGUUAUGUUACUAAUGUAUG |
| si*PAIP1* #1-2 | UACAUUAGUAACAUAACUCAA |
| si*DARS2* #1-1 | GGAAUGUGCUGACCUUCUAGA |
| si*DARS*2 #1-2 | UAGAAGGUCAGCACAUUCCAG |
| si*SLC35F5* #1-1 | GGAGAGAGAUGAAGGUCAAGA |
| si*SLC35F5* #1-2 | UUGACCUUCAUCUCUCUCCCU |
| si*P300* #1-1 | CCGGUGAACUCUCCUAUAA |
| siP300 #1-2 | UUAUAGGAGAGUUCACCGG |
| shCtrl | TTCTCCGAACGTGTCACGT |
| sh*STAT3* #1-1 | TGTTGTTGTTCTTAGACAA |
| sh*STAT3* #1-2 | TTGTCTAAGAACAACAACA |
| sh*ELF1* #1-1 | GGATGTTGCTGAAGAAGAA |
| sh*ELF1* #1-2 | TTCTTCTTCAGCAACATCC |
| sh*CD274* #1-1 | CTGACATTCATCTTCCGTTTA |
| sh*CD274* #1-2 | TAAACGGAAGATGAATGTCAG |

**Supplementary Table S4. List of antibodies used in this study.**

| **Antibody** | **Reactivity** | **Supplier** | **Application** | **Catalog number** | **Dilution** |
| --- | --- | --- | --- | --- | --- |
| NSUN2 | Human, Mouse | Proteintech | IF, IHC,  Western blotting | 66580-1-Ig | 1:200 (IF),  1:1,000 (IHC),  1:5,000 (Western blotting) |
| PD-L1 | Human, Mouse | Proteintech | IF, IHC,  Western blotting | 66248-1-Ig | 1:200 (IF),  1:5,000 (IHC),  1:1,000 (Western blotting) |
| NKp46 | Human, Mouse | Invitrogen | IF, IHC | PA5-102860 | 1:100 (IF),  1:200 (IHC) |
| NK1.1 | Mouse | Abcam | IF | ab242134 | 1:200 |
| CD8 | Mouse | Proteintech | IF | 29896-1-AP | 1:200 |
| Ki67 | Human, Mouse | Abcam | IHC | ab15580 | 1:200 |
| β-actin | Human | ABclonal | Western blotting | AC038 | 1:10,000 |
| ENO1 | Human | CST | Western blotting | 3810T | 1:1,000 |
| PKM | Human | Proteintech | Western blotting | 10078-2-AP | 1:1,000 |
| LDHA | Human | CST | Western blotting | #20125 | 1:1,000 |
| Pan-lactylation | Human | PTM | Western blotting | PTM-1401RM | 1:1,000 |
| H3K18la | Human | PTM | Western blotting, CUT&Tag-seq | PTM-1427RM | Western blotting (1:1,000), CUT&Tag-seq (1:100) |
| ELF1 | Human | CST | Western blotting | 31600T | 1:1,000 |
| H3K14la | Human | PTM | Western blotting | PTM-1414RM | 1:1,000 |
| H3K9la | Human | PTM | Western blotting | PTM-1419RM | 1:1,000 |
| Histone H3 | Human | PTM | Western blotting | PTM-1001RM | 1:1,000 |
| STAT3 | Human | CST | Western blotting | 9139S | 1:1,000 |
| pSTAT3 (Tyr705) | Human | CST | Western blotting | 9145S | 1:1,000 |
| P300 | Human | CST | Western blotting | 86377S | 1:1,000 |
| STAT3 | Human | CST | ChIP-seq, ChIP-qPCR | 9139S | ChIP-seq (1:100), ChIP-qPCR (1:100) |
| m^5^C | N/A | Active Motif | MeRIP-seq, MeRIP-qPCR | #39649 | 10 μg/IP |
| m^5^C | N/A | Abcam | Dot blot | ab214727 | 1:1,000 |
| IgG | N/A | Proteintech | negative control | 30000-0-AP | 1:100 |
| CD45 | Human | BioLegend | Flow cytometry | #982304 | 1:100 |
| CD3 / CD16+  CD56 cocktail | Human | BioLegend | Flow cytometry | #319101 | 1:100 |
| GZMB | Human | BioLegend | Flow cytometry | #515406 | 1:100 |
| IFNG | Human | BioLegend | Flow cytometry | #502526 | 1:100 |
| CD56 | Human | BioLegend | Flow cytometry | #985902 | 1:100 |
| PD-L1 | Human | BioLegend | Flow cytometry | #329707 | 1:100 |
| Annexin V | N/A | BD | Flow cytometry | #556419 | 1:100 |
| CD45 | Mouse | BioLegend | Flow cytometry | #103107 | 1:100 |
| CD3 | Mouse | BioLegend | Flow cytometry | #100235 | 1:100 |
| NK1.1 | Mouse | BioLegend | Flow cytometry | #108708 | 1:100 |

Abbreviations: IF, immunofluorescence; IHC, immunohistochemistry; WB, Western blotting; ChIP, chromatin immunoprecipitation; qPCR, quantitative polymerase chain reaction; CST, Cell Signaling Technology; PTM, PTM Biolabs; BD, BD Biosciences; IgG, immunoglobulin G; m^5^C, 5-methylcytosine.

**Supplementary Table S5. Sequences of primers used for ChIP-qPCR.**

| **Gene** | **Species** | **Forward primer** | **Reverse primer** |
| --- | --- | --- | --- |
| *CD274* | Human | CAAGGTGCGTTCAGATGTTG | GGCGTTGGACTTTCCTGA |

Abbreviations: ChIP, chromatin immunoprecipitation; qPCR, quantitative polymerase chain reaction; *CD274*, programmed cell death 1 ligand 1 gene.

**Supplementary Table S6. List of genes included in the NK signature.**

| **Symbol** | **Full name** | **Functional category** | **Key function** |
| --- | --- | --- | --- |
| *CTSW* | Cathepsin W | Cytotoxicity / Protease | Cysteine protease involved in immune response. |
| *GNLY* | Granulysin | Cytotoxicity | Antimicrobial and tumoricidal protein released by cytotoxic lymphocytes. |
| *GZMA* | Granzyme A | Cytotoxicity | Serine protease that induces caspase-independent cell death. |
| *GZMB* | Granzyme B | Cytotoxicity | Key apoptotic protease; primary mediator of NK cell cytotoxic activity. |
| *GZMH* | Granzyme H | Cytotoxicity | Serine protease with pro-apoptotic function in immune surveillance. |
| *IL2RB* | Interleukin 2 Receptor Subunit Beta | Signaling / Activation | Component of the high-affinity IL-2 receptor, critical for NK cell proliferation and activation. |
| *KLRB1 / CD161* | Killer Cell Lectin Like Receptor B1 | Co-stimulation / Adhesion | Binds to lectin-like ligands; modulates NK cell activation threshold. |
| *KLRC1 / NKG2A* | Killer Cell Lectin Like Receptor C1 | Inhibitory Receptor | Forms heterodimer with CD94; binds HLA-E and transmits inhibitory signal. |
| *KLRF1 / NKp80* | Killer Cell Lectin Like Receptor F1 | Activating Receptor | Activates NK cell cytotoxicity and cytokine production. |
| *KLRK1 / NKG2D* | Killer Cell Lectin Like Receptor K1 | Activating Receptor | Major activating receptor binding to stress-induced ligands. |
| *NCAM1 / CD56* | Neural Cell Adhesion Molecule 1 | Adhesion / Identity Marker | Classic surface marker defining human NK cells; mediates cell adhesion. |
| *NCR1 /*  *NKp46* | Natural Cytotoxicity Triggering Receptor 1 | Activating Receptor | Pivotal natural cytotoxicity receptor; specific marker for NK cell lineage. |
| *NKG7* | Natural Killer Cell Granule Protein 7 | Effector / Activation | Highly expressed in activated NK and T cells; essential for granule exocytosis. |
| *PRF1* | Perforin 1 | Cytotoxicity | Pore-forming protein facilitating granzyme entry into target cells. |

**Supplementary Table S7. List of 86 top-ranked candidate genes identified by CRISPR screening.**

| **Gene** symbol | **Negative r**ank | **Negative log**_2_FC | **RRA score** ( × 10^-5^) | **Negative-selection *P*** value |
| --- | --- | --- | --- | --- |
| *BCL7A* | 1 | -0.603 | 2.640 | <0.001 |
| *SIGLEC9* | 2 | -1.110 | 3.400 | <0.001 |
| *SRSF9* | 3 | -1.453 | 6.380 | <0.001 |
| *USP37* | 4 | -0.875 | 7.920 | <0.001 |
| *NAP1L4* | 5 | -1.322 | 9.180 | <0.001 |
| *NSUN2* | 6 | -0.925 | 10.592 | <0.001 |
| *OGT* | 7 | 0.014 | 13.193 | <0.001 |
| *MATK* | 8 | -1.309 | 13.695 | <0.001 |
| *DSE* | 9 | -1.801 | 17.760 | <0.001 |
| *KRT16* | 10 | -0.565 | 18.470 | <0.001 |
| *DIRC2* | 11 | -1.522 | 19.539 | <0.001 |
| *ECHS1* | 12 | -1.728 | 21.315 | <0.001 |
| *COX11* | 13 | -1.455 | 23.029 | <0.001 |
| *ADAMTSL3* | 14 | 0.468 | 23.747 | <0.001 |
| *CYB561* | 15 | -1.008 | 24.713 | <0.001 |
| *RAB41* | 16 | -0.964 | 25.201 | <0.001 |
| *RASA3* | 17 | -1.630 | 27.351 | <0.001 |
| *OR4M1* | 18 | -1.478 | 29.024 | <0.001 |
| *SLC17A2* | 19 | -1.081 | 34.300 | 0.001 |
| *SLC35G1* | 20 | -1.413 | 34.356 | 0.001 |
| *FNBP1L* | 21 | -1.146 | 36.773 | 0.001 |
| *FATE1* | 22 | 0.041 | 39.577 | 0.001 |
| *CLEC4G* | 23 | -0.682 | 44.853 | 0.001 |
| *HIAT1* | 24 | -1.195 | 48.054 | 0.001 |
| *SETDB1* | 25 | -0.947 | 49.388 | 0.001 |
| *ELOVL3* | 26 | -1.080 | 49.463 | 0.001 |
| *GTF2IP4* | 27 | -1.245 | 49.657 | 0.001 |
| *FAM134A* | 28 | 0.279 | 50.129 | 0.001 |
| *TEX2* | 29 | -0.589 | 55.405 | 0.002 |
| *TUBAL3* | 30 | -0.730 | 55.896 | 0.002 |
| *FARP1* | 31 | -1.436 | 58.132 | 0.002 |
| *GATA2* | 32 | -0.724 | 59.033 | 0.002 |
| *NYNRIN* | 33 | -1.266 | 60.154 | 0.002 |
| *BRI3BP* | 34 | -1.172 | 60.680 | 0.002 |
| *SMIM4* | 35 | -1.042 | 64.635 | 0.002 |
| *SLC35F5* | 36 | 0.448 | 65.956 | 0.002 |
| *PENK* | 37 | -0.866 | 67.505 | 0.002 |
| *PDHX* | 38 | -1.115 | 67.896 | 0.002 |
| *SORCS1* | 39 | 0.160 | 71.231 | 0.002 |
| *SMCO3* | 40 | -0.957 | 71.902 | 0.002 |
| *IGSF1* | 41 | -0.780 | 76.397 | 0.002 |
| *KRT19* | 42 | -1.007 | 76.506 | 0.002 |
| *ARHGAP24* | 43 | -0.851 | 80.962 | 0.002 |
| *CIZ1* | 44 | -0.557 | 81.781 | 0.002 |
| *CCDC167* | 45 | -1.533 | 86.097 | 0.002 |
| *ZNF691* | 46 | -1.561 | 87.055 | 0.002 |
| *DARS2* | 47 | -0.697 | 92.330 | 0.003 |
| *MAP4* | 48 | -1.489 | 93.220 | 0.003 |
| *PRKCA* | 49 | -0.760 | 96.253 | 0.003 |
| *ATXN7L2* | 50 | -0.460 | 97.604 | 0.003 |
| *PTBP3* | 51 | -1.372 | 101.390 | 0.003 |
| *CEBPZ* | 52 | -1.510 | 102.880 | 0.003 |
| *TMEM97* | 53 | -1.430 | 103.150 | 0.003 |
| *ATP5D* | 54 | -1.237 | 105.490 | 0.003 |
| *CDK5* | 55 | -0.927 | 108.010 | 0.003 |
| *FBXO3* | 56 | -1.022 | 108.150 | 0.003 |
| *PIP4K2C* | 57 | -1.062 | 108.400 | 0.003 |
| *GPR32* | 58 | -1.016 | 112.800 | 0.003 |
| *FAM217B* | 59 | 0.349 | 113.430 | 0.003 |
| *TENM3* | 60 | -0.975 | 113.810 | 0.003 |
| *CDCA5* | 61 | 0.180 | 118.700 | 0.003 |
| *HACD2* | 62 | -0.773 | 119.580 | 0.003 |
| *ZNF662* | 63 | -1.378 | 121.230 | 0.003 |
| *PAIP1* | 64 | 0.188 | 123.970 | 0.004 |
| *NUDT21* | 65 | -2.664 | 125.500 | 0.004 |
| *LRRC4B* | 66 | 0.145 | 129.250 | 0.004 |
| *NDST3* | 67 | -1.825 | 131.050 | 0.004 |
| *TANK* | 68 | -0.819 | 134.520 | 0.004 |
| *PSORS1C2* | 69 | 0.252 | 139.790 | 0.004 |
| *SSC5D* | 70 | -0.826 | 139.890 | 0.004 |
| *SLC12A1* | 71 | -1.659 | 145.060 | 0.004 |
| *PET112* | 72 | -1.454 | 150.340 | 0.004 |
| *EGLN3* | 73 | -0.556 | 155.610 | 0.004 |
| *TRAPPC5* | 74 | -0.764 | 156.700 | 0.004 |
| *GGTLC2* | 75 | -1.331 | 159.630 | 0.005 |
| *SKA3* | 76 | -1.206 | 160.000 | 0.005 |
| *C16orf46* | 77 | -1.333 | 160.830 | 0.005 |
| *SDK1* | 78 | -1.069 | 160.880 | 0.005 |
| *PUS7* | 79 | -0.957 | 161.810 | 0.005 |
| *TMEM221* | 80 | -1.104 | 162.900 | 0.005 |
| *SYTL2* | 81 | -0.090 | 166.150 | 0.005 |
| *LMO7* | 82 | -1.831 | 169.340 | 0.005 |
| *CLDN7* | 83 | -0.251 | 171.420 | 0.005 |
| *MED28* | 84 | -1.362 | 173.700 | 0.005 |
| *SAC3D1* | 85 | -0.079 | 176.700 | 0.005 |
| *FCGR2B* | 86 | -0.597 | 180.290 | 0.005 |

**Note:** The table lists candidate genes identified through negative selection analysis (using the MAGeCK algorithm). Columns indicate the gene symbol, negative rank, negative log_2_ fold change (log_2_FC), robust rank aggregation (RRA) score and negative-selection *P*-value for the NK cell co-culture group relative to the control.
